# Exciton‐Photon Coupling Microcavity as a Selective Biosensing Platform for Nonlocal Terahertz Metamaterials

**DOI:** 10.1002/advs.202416951

**Published:** 2025-02-28

**Authors:** Guifang Wu, Fengping Yan, Lanju Liang, Xuemei Du, Wei Wang, Ting Li, Junping Tian, Xin Yan, Haiyun Yao, Ziqun Wang, Meng Wang

**Affiliations:** ^1^ School of Electronic and Information Engineering Beijing Jiaotong University Beijing 100044 China; ^2^ School of Opto‐electronic Engineering Zaozhuang University Zaozhuang 277160 China; ^3^ Beijing Institute of Environmental Characteristics National Key Laboratory of Scattering and Radiation Beijing 100854 China; ^4^ School of Semiconductor and Physics North University of China Taiyuan 030051 China; ^5^ School of Physical Science and Engineering Beijing Jiaotong University Beijing 100044 China; ^6^ Department of Cardiovascular Medicine Beijing Tiantan Hospital Capital Medical University Beijing 100070 China

**Keywords:** anapole metamaterials, colloidal gold, exciton‐photon coupling microcavity, selective recognition

## Abstract

The strong‐coupling microcavity between excitons and photons facilitates efficient modulation and control of light, as well as precise manipulation of photon propagation properties. This phenomenon demonstrates significant potential for diverse applications in quantum information processing, optical sensing, and nonlinear optics. The anapole, as a specific type of captured state, allows for effective control over the electromagnetic field through appropriate distributions of current and charge, generating substantial localized effects within the field. This mechanism provides a novel avenue for investigating the strong‐coupling dynamics between photons and excitons in hybrid metamaterial sensing. Here, the rate of energy exchange between the excitons and the optical microcavity of the metamaterial is greater than their individual dissipation rates, resulting in significant Rabi splitting phenomena and pronounced anti‐crossing behavior, ultimately forming an “ultrasensitive photoreactive region” suitable for sensing applications. Furthermore, the nonlocal metamaterial, characterized by strong light‐matter coupling, can be integrated with functionalized colloidal gold and monoclonal tag antibodies to enable rapid multidimensional detection and identification of total prostate‐specific antigen (tPSA) in complex environmental solutions. The proposed strong‐coupling resonance microcavity plays a crucial role in the rapid evolution of nonlocal metamaterials, enhancing their applicability in molecular detection and selective recognition of fundamental light‐matter interaction phenomena.

## Graphical abstract

The nonlocal metamaterial, characterized by strong light‐matter coupling, can be integrated with functionalized colloidal gold and monoclonal tag antibodies to enable rapid multidimensional detection and identification of total prostate‐specific antigen in complex environmental solutions. The proposed strong‐coupling resonance microcavity plays a crucial role in the rapid evolution of nonlocal metamaterials, enhancing their applicability in molecular detection and selective recognition.

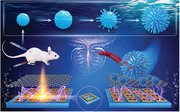

## 1 Introduction

Exciton‐polaritons are bosonic quasiparticles formed due to strong coupling between excitons (electron‐hole pairs) and cavity photons in a semiconductor microcavity.^[^ ^1^, ^2^, ^3^, ^4^ ^]^ The interaction between photons and excitons enters a state of strong coupling when the rate of energy exchange between the excitons and the optical microcavity is greater than their individual dissipation rates, resulting in Rabi splitting and anti‐crossover behavior in the spectra.^[^ ^5^, ^6^, ^7^ ^]^ Interestingly, when excitons are coupled to the resonant optical modes in the cavity, a continuous reversible energy exchange occurs, leading to light‐matter entanglement, and thus generating new hybrid light‐matter states.^[^ ^8^ ^]^ Additionally, the resultant exciton‐polaritons are quasiparticles with both exciton‐like and photon‐like characteristics — for example, high spatial coherence inherited from the photonic component and strong nonlinearities acquired from the excitonic counterpart.^[^ ^9^, ^10^, ^11^ ^]^ These remarkable properties make exciton‐polariton‐coupled cavities an ideal platform for the study of unusually fascinating quantum optical phenomena, including advanced optoelectronic applications such as slow‐light devices and inversionless polariton lasers. Particularly, the investigation of light‐matter interactions in 2D semiconductors is of fundamental importance for understanding many‐body effects and exploring their applications in photonics.^[^ ^12^, ^13^, ^14^, ^15^, ^16^ ^]^ The emerging family of 2D semiconductors with zero bandgaps, such as atomically thin black phosphorus, transition‐metal dichalcogenides, indium selenide, and graphene, are becoming promising active media for further developing 2D semiconductor microcavities. On the “matter” side, there are free, bound, and localized excitons in these 2D semiconductors, with binding energies ranging from tens to hundreds of meV.^[^ ^17^ ^]^ Furthermore, in the weak coupling regime, the interactions between excitons and photons in a microcavity can be controlled, enabling the realization of the Purcell effect and photon lasing. In the strong‐coupling regime, Rabi splitting and polariton lasing can be anticipated through the formation of polaritons and Bose‐Einstein condensates, respectively.^[^ ^18^, ^19^ ^]^ Based on a thorough investigation of the “light” and “matter” characteristics of 2D semiconductors and their microcavity devices, promising applications are expected in next‐generation coherent light sources, cavity‐enhanced single‐photon emitters, topological photonics, and other nonlinear optical devices. In the past, the strong coupling between excitons in semiconductors and optical microcavities relied primarily on the intervention of metal nanocavities supporting surface plasmon polaritons.^[^ ^20^ ^]^ This is because surface‐plasmon polaritons enable the optical field modes to be localized in a very small volume, which not only overcomes the diffraction limit, but also effectively enhances the local density of states of the electromagnetic wave, representing an optimum platform for achieving strong coupling. Yet metals generally possess high optical losses, especially in the terahertz (THz) band, where the transmission efficiency of photons at the metal interface is reduced due to the scattering and absorption effects of free electrons, thus affecting the photon‐matter coupling efficiency. However, an anapole, as a specific type of captured state, allows for effective control over the electromagnetic field through appropriate distributions of current and charge.^[^ ^21^, ^22^, ^23^, ^24^ ^]^ The anapole resonance exhibits localization and enhancement of the electromagnetic field without an accompanying energy loss of electromagnetic radiation, and it can significantly enhance the efficiency of the photon‐matter interaction, providing a novel approach to the investigation of the dynamics of the strong coupling between photons and excitons.

The development of miniaturization and integration has further facilitated the combination of coupling techniques with novel materials, such as nanomaterials and phase‐change materials, thus enhancing the efficiency and sensitivity of optical excitation.^[^ ^25^, ^26^ ^]^ However, the introduction of optical components adds complexity to the system, and high‐precision optical paths impose stricter requirements for stable operating conditions.^[^ ^12^ ^]^ Optical losses during signal transmission can lead to signal attenuation, so that scattering and absorption phenomena become more pronounced. The proposed platform for strong‐coupling resonance between excitons and photons plays a crucial role in the rapid evolution of nonlocal metamaterials. The structures of these hybrid metamaterials typically consist of subwavelength‐scale metals and dielectrics,^[^ ^27^, ^28^ ^]^ enabling the materials to exhibit unique electromagnetic properties at optical frequencies. In such materials, strong coupling between excitons and photons gives rise to a new quantum state known as the exciton‐photon mixed state, which not only enhances the excitation efficiency of excitons but also broadens their energy range, allowing them to operate over a broad range of the electromagnetic spectrum.^[^ ^8^ ^]^ Furthermore, the exciton‐photon strong‐coupling resonance microcavity leverages local effects, allowing light propagation to extend beyond localized regions and facilitating effective interactions over larger areas. This nonlocality enables excitons to exhibit enhanced integration capabilities under the influence of the photon field, aiding in the achievement of ultra‐high sensitivity in optical detection.^[^ ^3^, ^5^, ^20^ ^]^ For instance, in biosensing applications, the exciton‐photon strong coupling platform can be employed to detect extremely low concentrations of biomolecules by monitoring subtle shifts in the exciton‐photon resonance frequency, thereby enabling sensitive recognition of these biomolecules. Unfortunately, the strong interaction between the anapole resonance excited in metamaterials and excitons in semiconductor microcavities has not yet emerged as a promising approach for exciting research on strong exciton‐photon interactions. More specifically, there have been no proposals for its application in trace‐molecule detection in biosensors or for the selective recognition of analytes in complex solutions.

The exploration of exciton‐photon coupling within microcavities offers a transformative approach to biosensing applications, particularly through the innovative use of non‐local THz graphene metamaterials. Non‐local metamaterials, viewed as 2D structured optical films, can effectively manipulate radiative losses. They exhibit significant interactions between adjacent meta‐atoms and typically support well‐localized modes, which propagate transversely within the periodic structure plane. Moreover, this monolayer graphene is deposited atop the aluminum resonant units of the metamaterials. By modulating the chemical environment and electronic structure of monolayer graphene, the coupling efficiency between light and matter can be further refined. As reported below, we have experimentally conducted the study of hybrid metamaterial microcavities and observed significant Rabi splitting phenomena and pronounced anti‐crossing behavior, indicating that the system has reached a strong‐coupling state. This means that the energy exchange rate between excitons and photons has markedly intensified, leading to a system that exhibits exceptional sensitivity and responsiveness. Additionally, the integration of functionalized colloidal gold with monoclonal tag antibodies markedly enhances the efficacy of this platform. By harnessing the unique optical properties of colloidal gold, along with its selective capacity to capture target antigens, we successfully achieved multidimensional detection and selective identification of target antigens within complex environmental solutions. This dual‐functional design exemplifies the remarkable potential of employing strong light‐matter coupling mechanisms to advance high‐sensitivity biosensing and provides a novel pathway for the development of innovative quantum devices.

## 2 Strong Exciton‐Photon‐Coupled Microcavities in Hybrid Metamaterials

**Figure 1:**
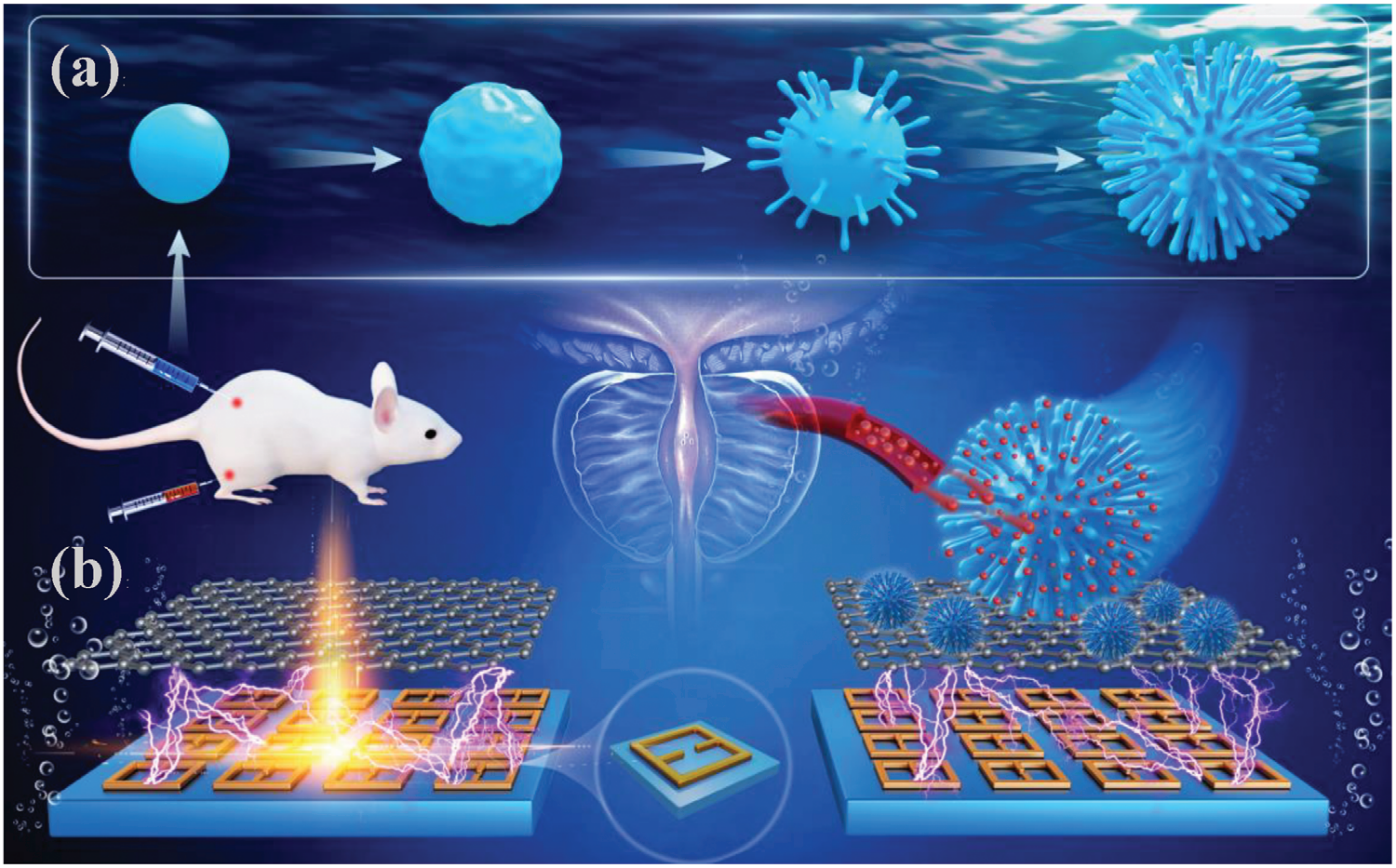
A schematic of the sensing strategy based on the proposed exciton‐photon‐coupled microcavity metamaterials. a) The schematic workflow of the colloidal gold labeled with target antibodies. b) The proposed monolayer graphene metamaterials, characterized by strong light‐matter coupling, are integrated with functionalized immune colloidal gold and monoclonal tag antibodies to facilitate the rapid detection and identification of target tPSA in complex environmental solutions. Inset: the proposed metamaterials are structured around a unit cell composed of two aluminum open rectangular resonant rings positioned on a polyimide substrate, featuring a periodicity of 120 µm.

Nanostructures exhibiting spatially varying optical properties have been extensively employed in wavefront‐manipulation applications, spatial‐frequency filtering, and refractive‐index sensing over a broad range of the electromagnetic spectrum. The metamaterials proposed here are structured around a unit cell composed of two aluminum open rectangular resonant rings positioned on a polyimide substrate, featuring a periodicity of 120 µm, as illustrated schematically in **Figure** **1**. The detailed fabrication methods for the metamaterials are described in Section S1 (Supporting Information). Given that exciton‐polaritons arise from strong‐coupling phenomena, monolayer graphene — with its zero bandgap and elevated transition dipole moment — emerges as an optimal material for enhancing the coupling strength between light and matter.^[^ ^19^, ^20^ ^]^ This monolayer graphene is deposited atop the aluminum resonant units of the metamaterials. Moreover, by modulating the chemical environment and electronic structure of monolayer graphene, the coupling efficiency between light and matter can be further refined, as depicted in Section S2 (Supporting Information), thereby augmenting its potential applications in nonlocal metamaterial sensors. Additionally, the proposed metamaterials, characterized by strong light‐matter coupling, are integrated with functionalized immune colloidal gold and monoclonal tag antibodies to facilitate the rapid detection and identification of total prostate‐specific antigen (tPSA) in complex environmental solutions. Notably, the colloidal gold can covalently conjugate to primary amines (‐NH_2_) of proteins,^[^ ^29^, ^30^, ^31^, ^32^, ^33^ ^]^ thereby enhancing the capability of the immunosensor to detect these proteins. Figure 1a shows the schematic workflow of modifying colloidal gold labeled with target antibodies for selective binding to tPSA. The detailed process of functionalized colloidal gold binding to the antibody and target tPSA, along with scanning electron microscope images, can be found in Sections S2 and S3 (Supporting Information). Hence, through the innovative design and integration of materials, this research not only provides new insights into the development of local immunosensors but also opens new avenues for other early diagnostic technologies in the biomedical field.

**Figure 2:**
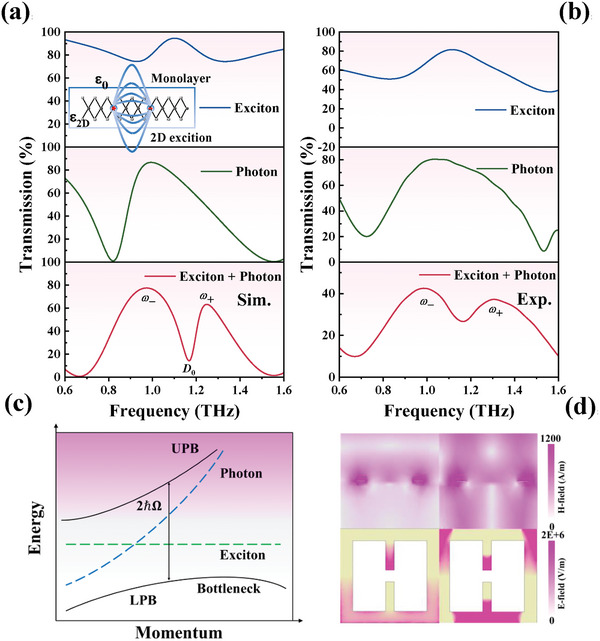
Fundamentals and mechanism analysis of the interaction in strong exciton‐photon coupling cavities. a) calculated. b) measured. Blue solid lines: the prominent transmission peak appears in the continuous spectrum of monolayer graphene; Green solid lines: the transmission spectrum of the proposed metamaterial based on non‐radiating anapole resonance; Red solid lines: the energy states of excitons and photons transition from being simple independent states to forming hybrid states, revealing a pronounced Rabi splitting phenomenon. Inset: This peak arises from the formation of excitons, which are bound states created by the Coulomb interaction between electrons and holes.^[^ ^15^ ^]^ c) A dispersion plot of exciton‐photon (black solid lines) that includes LPB and UPB. The blue dotted line represents the cavity photon dispersion and the green dashed line represents the exciton dispersion, respectively. The black solid arrow marks the Rabi splitting energy. Reproduced with permission from ref. [13, 14] d) The near‐field intensity at the peak is significantly greater than that of the metamaterial based on non‐radiating anapole resonance at the same frequency.

The strong coupling between excitons and photons in the proposed metamaterial system is a direct result of the interaction between two fundamental quantum systems: the light (photon) field and the matter (exciton) field. This coupling is of paramount importance for various applications in quantum optics, sensing, and material design. Excitons, which are bound states of an electron and a hole created by Coulomb attraction, are particularly pronounced in materials with low‐dimensional structures like monolayer graphene. As illustrated in **Figure** **2**, this work presents numerical simulations and experimental validations indicating that a prominent transmission peak appears in the continuous spectrum of monolayer graphene. The unique electronic band structure of monolayer graphene enables a high density of states near the Dirac point, thereby facilitating the formation of excitons, particularly under specific conditions such as optical excitation or the presence of external fields.^[^ ^17^ ^]^ Moreover, owing to the layer's small thickness (≈1 nm), electrons and holes are strongly confined in a quantum regime, and their wave functions have large overlap, naturally forming strongly bound excitons even at room temperature,^[^ ^19^ ^]^ as depicted in Figure 2a. When monolayer graphene is integrated with the aluminum‐based metamaterial, the coupling between photons and excitons is enhanced through the resonant interaction facilitated by the electromagnetic field. The metamaterial structure, specifically the open rectangular resonant rings, facilitates strong localized electromagnetic fields at certain frequencies, allowing photons in these localized modes to couple effectively with excitons. Furthermore, to better elucidate the mechanism of the resonance generated by the proposed metamaterials without monolayer graphene, the scattering powers of various multipoles were computed based on the spatial current density distribution in Cartesian coordinates,^[^ ^21^, ^22^ ^]^ as depicted in Figure S3 (Supporting Information) (see Section S4, Supporting Information). The contributions of toroidal dipole (TD) and electric dipole (ED) predominantly govern the far‐field scattered power from the metamaterials at 0.98 THz. Simultaneously, ED and TD exhibit opposite phases, with a phase difference of π/2, such that destructive interference between TD and ED leads to a non‐radiating anapole resonance. This anapole principally exhibits the localization and enhancement of the electromagnetic field without an accompanying energy loss of electromagnetic radiation, and it can significantly enhance the efficiency of strong coupling with the previously mentioned excitons. Nevertheless, the prerequisite for strong coupling to appear is that the photon and exciton modes must overlap, encompassing both their near‐field interactions and the alignment of their spectral positions. Subsequently, by adding monolayer graphene as the active material on the metamaterial, the interaction between excitons (bound states of electrons and holes) and photons (quanta of the electromagnetic field) is influenced by the coupling strength. Also, the energy states of excitons and photons transition from being simple independent states to forming hybrid states, resulting in energy‐level interleaving rather than mere crossing (Figure 2a–c). The new energy states behave as two separated energy branches — specifically, two distinct transmission peaks (upper polariton branch (UPB) and lower polariton branch (LPB)), which are “anti‐crossing” in energy–momentum space. The minimum energy difference between the two branches is called the Rabi‐splitting energy (referred to as 2ℏΩ^[^ ^7^, ^14^ ^]^ or ℏΩ^[^ ^19^ ^]^), and this behavior is also manifested in the transmission spectrum of the hybrid metamaterial, revealing a pronounced Rabi splitting phenomenon (Figure 2a,b). In general, light‐matter interactions within semiconductor metamaterial microcavities can be classified into weak and strong coupling regimes by analyzing the relationships of the coupling strength of *g* with the decay rates of bare excitons (*γ*) and cavity photons (*κ*), where *γ* and *κ* can be experimentally or numerically approximated by measuring the half‐line widths of bare photon and exciton modes.^[^ ^4^, ^6^, ^9^ ^]^ For the quantum boxes or dots in semiconductor metamaterial microcavities, the weak and strong coupling interactions are defined by g ≤ |γ − κ|/2 and g   >   |γ − κ|/2, respectively.^[^ ^17^ ^]^ To clearly visualize the polariton mode splitting in 2D‐semiconductor metamaterial coupled photonic structures, the strong coupling condition of exciton‐photon interactions is raised to or Rabi‐splitting energy 2ℏΩ = 2(*g* ^2^ − 0.25(γ − κ)^2^)^0.5^ > γ + κ.^[^ ^14^, ^17^, ^18^ ^]^ In Figure 2b, the experimental data are fitted to obtain a coupling strength of 0.705 meV, with values of *γ* = 0.39 meV, *κ* = 0.875 meV, and 2ℏΩ = 1.323 meV (the derivation is described in Section S5, Supporting Information). These results are in substantial agreement with those derived from direct fitting of the simulated spectra, indicating that the exciton‐photon coupling falls within the strong‐coupling regime. In the experiment, the transmission curve of the exciton‐photon hybrid cavity metamaterial is only half of that observed in the simulation. This discrepancy can primarily be attributed to the significant radiation losses caused by the high carrier concentration and conductivity of graphene. These radiation losses are more pronounced in the experiment than in the idealized model used in the simulation, resulting in further attenuation of the transmission signal. Additionally, for split‐ring resonators (SRRs) with small capacitive gaps, placing graphene on top may create conductive pathways, leading to short‐circuit effects. This short‐circuiting weakens the resonance strength of the SRRs by reducing the effective localization and enhancement of the electric field, ultimately causing energy dissipation in the resonance. Notably, the electromagnetic field density in the *xy* plane illustrates the localized near‐field intensity at the capacitive gaps and the surfaces of the rectangular resonant units, and the near‐field intensity at the peak (denoted as *ω_+_*, with *ω_+_* = 1.245 THz) is significantly greater than that of the metamaterial based on non‐radiating anapole resonance at the same frequency, as depicted in Figure 2d. This finding indicates a significant alteration in the energy levels of the coupled system, further validating the importance of near‐field enhancement effects in light‐matter interactions. Moreover, this near‐field enhancement not only increases the likelihood of exciton generation but may also facilitate the effective absorption of photons, thereby enhancing the overall efficiency of light‐matter coupling.

## 3 Experimental Demonstration of Selective Recognition and Detection of tPSA

**Figure 3:**
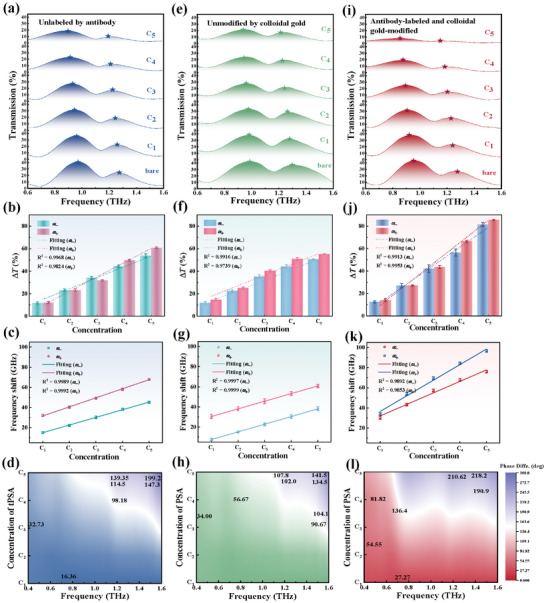
The transmission spectra, amplitude modulation depths, frequency shifts, and phase differences of the biosensors—those not labeled with anti‐tPSA, those not modified with colloidal gold, and those labeled and modified with anti‐tPSA and colloidal gold (collectively referred to as the immunosensor)—in detecting different concentrations of tPSA proteins. (a–d): The biosensor is unlabeled with anti‐tPSA. a) Normalized THz transmission spectra. b) The relationship between the modulation depth (Δ*T*) and concentration (*C_i_*) exhibits a pronounced linear correlation (R^2^ > 0.98). c) the frequency shifts (Δ*f*). d) To characterize the modulation and sensing performance at different concentrations quantitatively, Δ*P* = *P_0_* − *P_i_* (*P_0_* and *P_i_* are values of phases at bare and C_i_, respectively) of the corresponding concentration are calculated. (e–h): The biosensor is unmodified with colloidal gold. e) the transmission spectra. f) Δ*T*. g) Δ*f*. h) Δ*P*. (i‐l): The immunosensor. i) The experimentally measured transmission amplitude spectra of the immunosensor, reveal a steady decline in amplitude as the tPSA concentration increases from C_1_ to C_5_. j) At the coupling frequency points (ω‐) and (ω+), the maximum modulation depths reach ∆*T_max_* (ω_‐_) = 81.5% and ∆*T_max_* (ω_+_) = 85.4%, respectively. k) The coupling frequencies experience a more pronounced redshift, where the maximum shifts of *ω_−_* and *ω_+_* are 79 and 99.6 GHz, respectively, at a concentration of C_5_. (l) The hybrid metamaterial immunosensor achieves a phase difference of 210.62° at the coupling frequency point (ω_+_ = 1.17 THz).

The qualitative evidence from the above analysis demonstrates that, with the broad and intense light‐matter interactions enabled by the exciton‐photon coupled microcavity, the proposed hybrid non‐local metamaterial serves as an optimal platform for manipulating light and achieving ultrasensitive biochemical molecular sensing. tPSA, a glycoprotein produced by prostate cells, is typically generated in greater quantities by prostate cancer cells than by normal prostate cells, leading to markedly elevated tPSA levels in the bloodstream in the presence of prostate cancer. However, benign prostatic hyperplasia (BPH), prostatitis, and other non‐cancerous conditions can also cause elevated tPSA levels.^[^ ^34^, ^35^ ^]^ Consequently, the detection of tPSA protein concentration is crucial for the diagnosis of these diseases. In this work, five different concentrations of target tPSA protein solutions were tested: 10 fg mL^−1^ (C_1_), 1 pg mL^−1^ (C_2_), 100 pg mL^−1^ (C_3_), 10 ng mL^−1^ (C_4_), and 1 µg mL^−1^ (C_5_). The preparation and measurement methods for the various target tPSA protein concentrations are provided in Section S7 (Supporting Information). **Figure** **3**a illustrates the detection results of the monolayer graphene‐colloidal gold biosensor without modified anti‐tPSA on the surface. The target tPSA proteins at varying concentrations were dropped directly onto the hybrid metamaterial. Notably, after the introduction of colloidal gold, the spectrum of the coupled system still exhibits a pronounced Rabi splitting. However, due to the effects of localized surface plasmon resonance, the photon field undergoes redistribution, leading to a reconfiguration of the original photonic modes. This reconfiguration competes with or disrupts the exciton‐photon coupling effect, ultimately resulting in the weakening of the Rabi splitting (2ℏΩ = 1.262 meV, which is obtained by fitting the curve labeled “bare” in Figure 3a according to the Lorentz‐Drude model) and the coupling strength (*g* = 0.668 meV). Fortunately, this result indicates that the exciton‐photon coupling remains in the strong coupling regime at this point (g >[(γ^2^ + κ^2^)/2]^0.5^ = 0.582 meV), which suggests that the total transmission spectrum of the coupled system becomes dominated by the spectral features of the exciton‐photon hybrid metamaterial. As the concentration of the target tPSA protein increases, the enhancement of the electric field exacerbates energy loss in the THz waves, leading to a higher likelihood of these waves being reflected as they traverse the metamaterial, which is directly manifested as a significant reduction in transmittance. The transmission coefficient variation of the monolayer graphene‐colloidal gold biosensor at different concentrations, C_1_ to C_5_, is defined as ∆*T =* (*T_Ci_ – T_bare_*)%, where *T_Ci_* (*T_bare_*) is the transmission at the coupling frequency point with (without) the target tPSA protein. The maximum ∆*T* was reached at a concentration of 1 µg mL^−1^ (C_5_), where ∆*T_max_* (*ω_‐_*) = 53.3% and ∆*T_max_* (*ω_+_*) = 60.2% (Figure 3b), enabling ultra‐sensitive detection. This phenomenon is further attributed to the optical properties of monolayer graphene, which are closely tied to the surface carrier concentration: As the concentration increases, the scattering rate of carriers in monolayer graphene may rise, leading to enhanced absorption of THz waves and further reduction in transmittance. Most notably, as the target tPSA protein concentration increases, the changes in carrier concentration in monolayer graphene alter the interaction between excitons and photons, resulting in a shift in exciton energy. Experimentally, a blueshift of the G peak was observed (see Figure S5 and Section S8, Supporting Information), indicating that the Fermi level of monolayer graphene moved away from the Dirac point, and p‐type doping was further enhanced, thereby influencing the transmittance of THz waves.^[^ ^20^ ^]^

At the same time, the increased concentration of target tPSA protein may weaken the exciton binding energy, which in turn reduces the efficiency of exciton‐photon coupling, further impacting the degree and position of the Rabi peak splitting. This suggests a reconfiguration of the coupling mechanism between exciton behavior in monolayer graphene and photon states. The coupling frequencies of excitons and photons (ω_–_ and ω_+_) undergo a redshift (Figure 3c), and the relationship between the frequency shift (Δ*f*) and concentration (*C_i_*) exhibits a pronounced linear correlation (R^2^ > 0.99), which is consistent with a fitting function (green or red curve). The observed linearity arises from the possibility of multiple adsorbed molecules on the biosensor, leading to interference effects (when concentration increases) and hence with each additional molecule having an increasing impact on the THz frequency shift. Therefore, changes in the Rabi peaks not only reflect the optical properties of the metamaterial but also provide new insights into the electronic characteristics of graphene and exciton dynamics. Moreover, the transmission amplitude and phase through a structured surface are inherently interdependent, according to the Kramers–Kronig (KK) relation,^[^ ^36^, ^37^ ^]^ so that a stronger resonant intensity corresponds to a more substantial phase‐jump change near the resonant frequency —, i.e., the strong resonant intensity results in a large value of *dln*|*G*|/*dln*|ω| (the derivation is provided in Section S10, Supporting Information). The phase difference of the monolayer graphene hybrid metamaterial at the coupling frequency point (*ω_+_* = 1.20 THz) can reach 139.35° (Figure 3d). These findings indicate that our exciton‐photon coupling microcavity‐based biosensing platform exhibits high sensitivity and accuracy in measuring tPSA protein concentrations within the tested range of 10 fg mL^−1^ to 1 µg mL^−1^ in serum samples spiked with tPSA.

The intricate balance of repeated energy transfer between excitons and photons exhibits an exceptional sensitivity to external environmental doping disturbances; the aforementioned qualitative and quantitative analyses further validate the existence of this remarkable “ultrasensitive photoreactive region.” Nonetheless, to facilitate rapid multidimensional detection and identification of target proteins in complex environmental solutions, the non‐local hybrid metamaterial can be integrated with immune colloidal gold and labeled anti‐tPSA, resulting in a novel biosensor with selective recognition capabilities. Immune colloidal gold is distinguished by its excellent biocompatibility and ease of modification, rendering it effective for enhancing antibody binding and preserving its activity. The biosensor is initially activated by 3‐aminopropyltriethoxysilane (APTES), where the amine groups (‐NH_2_) in APTES react with the thiol groups (‐SH) or other reactive groups on the surface of the immune colloidal gold, forming a stable covalent bond.^[^ ^38^, ^39^, ^40^ ^]^ This covalent bond not only enhances the immobilization of antibodies on the surface of the biosensor but also substantially improves the efficiency of binding to the target molecule. The experimentally measured transmission amplitude spectra of tPSA protein tagged with anti‐tPSA in colloidal gold integrated with the hybrid metamaterial biosensor reveal a steady decline in amplitude as the tPSA concentration increases from C_1_ to C_5_, as illustrated in Figure 3i. In other words, the presence of functionalized colloidal gold conjugated with tag antibodies significantly boosts the target tPSA protein capture efficiency, and the modulation of THz waves by the exciton‐photon coupled microcavity metamaterial exhibits enhancement over a wide frequency range — from 0.6 to 1.6 THz — which can be compared with the monolayer graphene metamaterial without functionalized colloidal gold (Figure 3e). Figure 3j illustrates the modulation depth of the transmission coefficient of THz waves by the immunosensor when detecting different tPSA concentrations. At the coupling frequency points (ω_‐_ and ω_+_), the maximum modulation depths reach ∆*T_max_* (*ω_‐_*) = 81.5% and ∆*T_max_* (*ω_+_*) = 85.4%, — satisfying results. This modulation performance at the resonance peaks surpasses that of several graphene/GaN metamaterials,^[^ ^22^, ^27^, ^36^ ^]^ even when those metamaterials are excited by voltage, pump light, or thermal signals. Meanwhile, after labeling with the target antibody (notably, the concentration of anti‐tPSA (20 µg mL^−1^) is markedly higher than that of target tPSA protein to ensure adequate binding), the static doping effect in monolayer graphene becomes increasingly pronounced as target tPSA protein concentration rises. This phenomenon affects both the carrier concentration and the band structure of monolayer graphene. More critically, the substantial adsorption of analyte molecules onto the metamaterial surface enhances the electron shielding effect, while molecular scattering and absorption increase significantly. These combined factors hinder efficient coupling between excitons and the photon microcavity.

The variation in the Rabi splitting value is also affected by the energy difference between the excited and ground states of the excitons. As the target tPSA protein concentration increases, environmental changes in the metamaterial alter the exciton density of states, resulting in a gradual decrease in the splitting value 2ℏΩ and the coupling strength *g* until g < [(γ^2^ + κ^2^)/2]^0.5^, indicating that the coupling system undergoes a transition from strong coupling to weak coupling, as depicted in Figure S8 (Supporting Information) (Section S11, Supporting Information). Consequently, the coupling frequencies experience a more pronounced redshift (Figure 3k). The maximum shifts of *ω_−_* and *ω_+_* are 79 and 99.6 GHz, respectively, at a concentration of C_5_, which represents a significantly larger frequency shift than for the hybrid metamaterial without functionalized colloidal gold modification (Figure 3g) or without labeling the target antibodies (Figure 3c). Additionally, we can employ modified perturbation theory to elucidate the alterations in coupling frequency (the relationship between the change in dielectric environment and angular frequency shift is fully derived in Section S12, Supporting Information). Considering the exponential decay of the electric field along the direction normal to the hybrid metamaterial, the calculation of angular frequency shift is directly proportional to the concentration of the target antigen, underscoring the substantial impact of concentration variations on the response of the exciton‐photon‐coupled microcavities system. This correlation suggests that as the analyte concentration increases, the influence of the electric field becomes stronger, resulting in more significant frequency shifts, thereby further clarifying the intrinsic link between concentration and system characteristics. Additionally, the optical properties of colloidal gold, characterized by strong light scattering and absorption across the visible and THz frequency ranges, play a crucial role in enhancing the capture efficiency.^[^ ^30^, ^31^, ^32^, ^33^ ^]^ This enables colloidal gold‐based immunosensors to achieve significant amplification of molecular signals during detection, which permits efficient and multidimensional detection even at lower concentrations of target tPSA protein (C_1_ = 10 fg mL^−1^), as demonstrated in Figure 3i–l. As a result of the alteration in the coupling mechanism between photons and excitons, the amplification of the local electric field at the surface of the immunosensors directly influences the propagation characteristics of light, ultimately leading to a substantial enhancement of the phase variation of the THz waves —, i.e., as the concentration increases, the hybrid metamaterial immunosensor achieves a phase difference of 210.6° at the coupling frequency point (*ω_+_* = 1.17 THz). Additionally, we detected spiked target tPSA protein using ELISA (enzyme‐linked immunosorbent assay) and found that the sensitivity and detection limit of the exciton‐photon coupling microcavity metamaterial platform for protein detection were markedly superior to those of the ELISA gold standard method (see Section S13 and Figure S9, Supporting Information).

**Figure 4:**
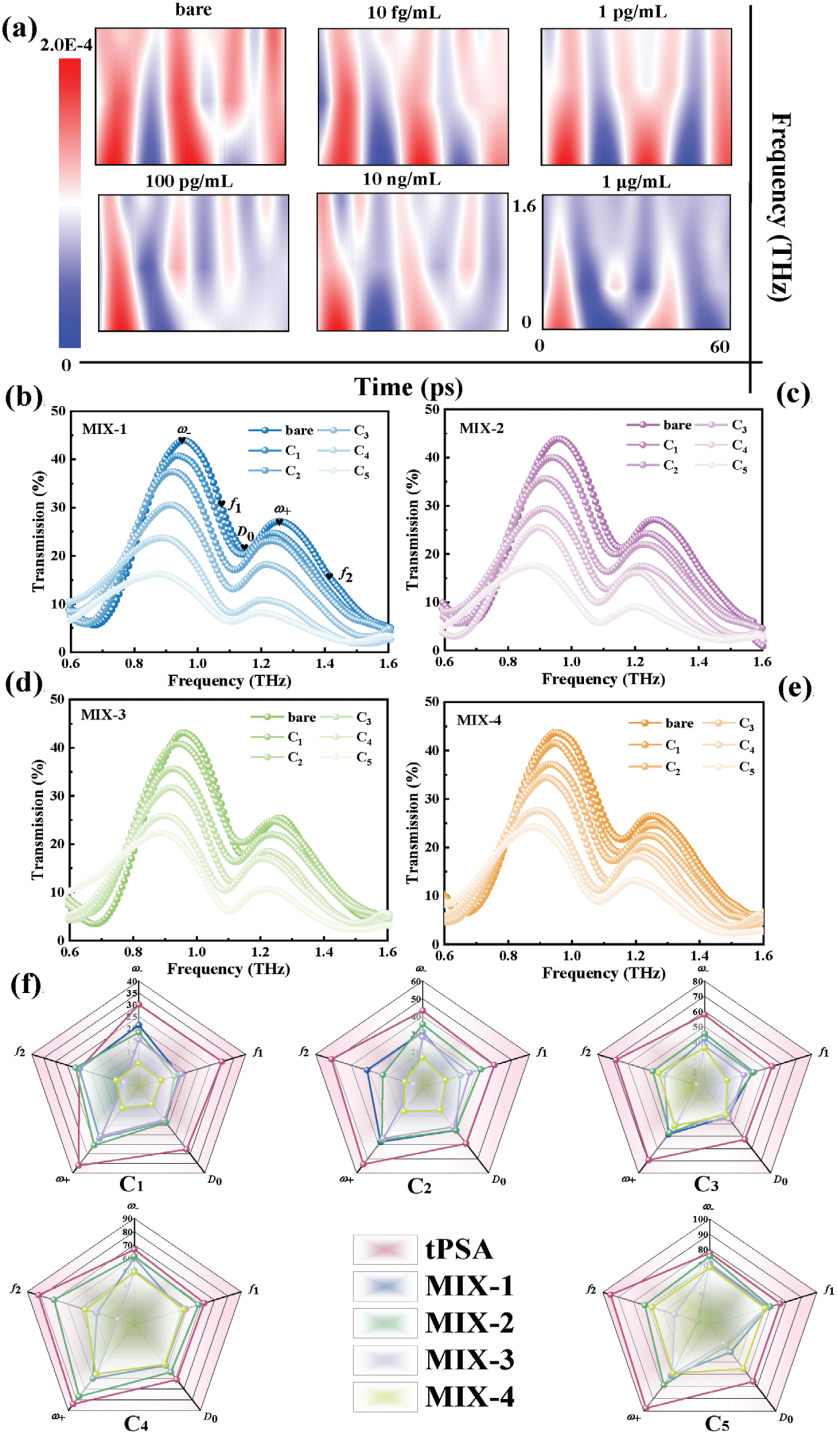
Specificity of the THz immunosensor for detecting target tPSA protein. a) The 2D extinction intensity plots of the target tPSA protein at each concentration can be used as standard graphic cards to differentiate between them as well as the solution concentrations. The normalized transmission spectra of the THz hybrid immunosensor when detecting different types of mixed samples. b) MIX‐1. c) MIX‐2. d) MIX‐3. e) MIX‐4. MIX‐1, comprising α‐Lactalbumin, D‐Threonine, and tPSA; MIX‐2, containing L‐Arginine, D‐Threonine, and tPSA; MIX‐3, a blend of D‐Threonine and L‐Arginine; and MIX‐4, a mixture of α‐lactalbumin and L‐Arginine. The frequency shifts and amplitude changes observed for MIXes 1 and 2 for target tPSA protein samples were markedly greater than those for MIXes 3 and 4 f) The frequency shifts of the four mixed samples and target tPSA protein at varying concentrations across three resonance points and two frequency points, forming a closed radar chart in the shape of a pentagram.

Additionally, to illuminate the interaction between THz waves and the immunosensors at varying concentrations of target tPSA protein with clarity and comprehensiveness, we propose a novel methodology that employs continuous wavelet transform (CWT) in conjunction with the convolution of Morlet wavelets applied in THz time‐domain spectroscopy (the derivation is described in Section S14, Supporting Information). This approach enables the rapid identification of trends in concentration variation. The two‐variable function CWT (a,b)^[^ ^41^, ^42^, ^43^ ^]^ effectively illustrates how the responses of different frequencies within the signal evolve over time, and by adjusting the scale parameter *a* and the position parameter *b*, we generate 2D wavelet‐coefficient‐intensity cards that visually convey the impact of varying tPSA concentrations on the transmission characteristics of THz waves, as depicted in **Figure** **4**a. The transmission amplitude of THz waves gradually diminishes as the concentration increases; this change is evident in the frequency domain and also highlights the propagation characteristics of THz waves in the time domain, along with the significant impact of the effects of coupling with the immunosensor within a specific time window (0 to 60 ps). This dynamic process is clearly represented in the time‐frequency domain, revealing the profound influence of concentration changes on the characteristics of THz waves. In addition, the immunosensor produces colorimetric standard cards for detecting varying concentrations, facilitating both qualitative and quantitative analyses of the target antigen. This capability significantly outperforms traditional colloidal gold techniques, which are limited to qualitative detection.

In biosensing applications, it is essential to evaluate the stability and selective detection capabilities of sensors for target biomolecules, given their significant implications for biological diagnosis and disease detection. Accordingly, in this work, grounded in the verification of an “ultrasensitive photoreactive region” within exciton‐photon microcavity hybrid metamaterials, we devised and conducted a series of control experiments to assess selective recognition ability of the immunosensor for target tPSA protein. We investigated four distinct mixed solutions: MIX‐1, comprising α‐Lactalbumin, D‐Threonine, and tPSA; MIX‐2, containing L‐Arginine, D‐Threonine, and tPSA; MIX‐3, a blend of D‐Threonine and L‐Arginine; and MIX‐4, a mixture of α‐lactalbumin and L‐Arginine. The primary distinction between MIXes 1 and 2 and MIXes 3 and 4 lies in the inclusion of the target‐marked tPSA protein in the former. Through a comparative analysis of the transmission spectra of these various mixed solutions (Figure 4b–e), we found that the amplitude of the transmission curves exhibits a more pronounced decay with increasing concentration, enabling detection at femtogram‐level concentrations, which underscores the stability of the detection sensitivity of the immunosensor across diverse mixed backgrounds.

Notably, the frequency shifts and amplitude changes observed for MIXes 1 and 2 (Figure 4b,c) were markedly greater than those for MIXes 3 and 4 (Figure 4d,e). This observation indicates that the other samples did not react with the anti‐tPSA and, consequently, were unable to bind to the functionalized colloidal gold. Although the concentrations of these samples are equivalent to that of the target tPSA protein, only a limited number of sample molecules manage to interact with the immunosensor's surface. Thus, it is evident that the metamaterial immunosensor demonstrates a highly sensitive electromagnetic response to tPSA, which affirms its capability for selective recognition of the target tPSA protein. Figure 4f illustrates the frequency shifts of the four mixed samples and target tPSA protein at varying concentrations across three resonance points and two frequency points, forming a closed radar chart in the shape of a pentagon. The results compellingly indicate that at a concentration of 1 µg mL^−1^ (C_5_) the area of the closed shape is maximized, with the target tPSA protein samples exhibiting the largest closed area at each concentration, followed by MIXes 1 and 2, while MIXes 3 and 4 exhibit the smallest areas. This suggests that researchers can effectively distinguish and identify the target tPSA in mixed samples solely by the pronounced differences in the area of the closed shapes in the radar chart, all without the necessity for any cell‐staining processes. Moreover, it is worth noting that when this immunosensor is used to detect varying concentrations of mixed or tPSA samples, the magnitude of the frequency shifts in the splitting peaks (i.e., *ω_–_* and *ω_+_*) consistently surpasses that observed at *D_0_*, even with concentration fluctuations. The behavior persists in the biosensors that are unmarked with anti‐tPSA (Figure 3a) or unmodified with colloidal gold (Figure 3e). In general, as the frequency increases, the interaction between the motion and vibration modes of the analyte molecules and the electromagnetic properties of the metamaterial become more pronounced. Also worth noting is that high‐frequency measurements, by involving shorter wavelengths, amplify the surface and local field enhancement effects of the mixed metamaterial on smaller scales, consequently producing more substantial frequency shifts (theoretically, the frequency shift at *D_0_* should be greater than that at ω). This “contradictory phenomenon” can be attributed to the pronounced electric dipole oscillation that materializes within the structural units at *D_0_* (as evidenced in Figure S3d, Supporting Information, where the electric dipole moment dominates). The electric field induced by the electric dipole resonance mode is concentrated primarily within the internal domain of the metamaterial unit, rendering it relatively insensitive to external environmental changes. Conversely, the “ultrasensitive photoreactive region” generated by the exciton‐photon coupling microcavity markedly enhances the probability of the immunosensor capturing trace molecules and effectively promotes the coupling of light field energy with sample molecules. Overall, the information derived from transmission amplitude, frequency shift, phase shift, 2D intensity maps, and pentagonal radar charts constitutes a collective multidimensional biosensing framework originating from the exciton‐photon microcavity metamaterials, thereby equipping researchers with multiple detection metrics that enhance both sensitivity and accuracy in analyzing the samples under investigation. To facilitate a comprehensive performance comparison with other existing immunosensors, the limit of detection (LOD), transmission amplitude, frequency shift, and phase shift of the hybrid metamaterial immunosensor designed in this study were compared with those of similar sensors reported in the literature. The results of this comparison, presented in **Table** **1**, clearly demonstrate that the designed immunosensor outperforms its counterparts in all four parameters. The improved performance is attributed to the unique properties of the hybrid metamaterials, which enhance light‐matter interactions and facilitate more efficient signal transduction. As a result, this immunosensor holds significant promise for future applications in diagnostic tools, environmental monitoring, and other fields requiring precise and sensitive detection capabilities.

**Table 1:** Comparing the performance with previous work using THz metamaterial immunosensor.

Immunosensors	LOD	∆*f_max_*	∆*T_max_*	∆*P_max_*
[29]	20 µg mL^−1^	87.5 GHz	N/A	N/A
[30]	100 ng mL^−1^	15.7 GHz	N/A	N/A
[31]	380 fM	N/A	N/A	N/A
[32]	10 pg mL^−1^	47.3 GHz	N/A	N/A
[33]	4.78 × 10^2^ CFU mL^−1^	27.21 GHz	N/A	N/A
This work	10 fg mL^−1^	99.6 GHz	85.4%	210.6°

LOD: the limit of detection. ∆*f_max_*: maximum change in frequency shift. ∆*T_max_*: maximum change in modulation depth. ∆*P_max_*: maximum change in phase shift.

## 4 Conclusions and Perspectives

In summary, we investigated, both numerically and experimentally, the complex balance of repeated energy transfer between excitons and photons, driven by the hybrid Rabi splitting mode, introduces new tunable features that exhibit ultra‐high sensitivity to external environmental doping disturbances, ultimately resulting in the formation of a striking “ultrasensitive photoreactive region.” Additionally, by integrating functionalized colloidal gold with monoclonal tag antibodies, we fabricated a nonlocal hybrid metamaterial that enables rapid detection and identification of target tPSA protein in complex environmental solutions. This design effectively leverages the unique optical properties of colloidal gold and the exceptional specificity of monoclonal antibodies, ensuring efficient capture of target antigens in complex biological samples. This approach — that is, the combination of an exciton‐photon‐coupled microcavity with the functionalized colloidal gold metamaterial immunosensor — will open up promising prospects for the detection of ultra‐low concentrations of molecules as well as for selective recognition mechanisms based on the phenomenon of light‐matter interactions. In further work, we expect to address the following challenges: 1) Although this hybrid immunosensor successfully enables the selective recognition of target molecules, the introduction of colloidal gold adversely affects the coupling characteristics prior to the detection of tPSA proteins. There is a pressing need to address the challenge of designing a nonlocal THz metamaterial biosensor that can selectively detect target molecules at femtogram‐level concentrations while enhancing the exciton‐photon coupling effect. 2) The exciton‐photon microcavity not only demonstrates exceptional sensitivity to molecular signals but may also exhibit pronounced responsiveness to optoelectronic and temperature signals. This attribute holds significant potential for propelling the advancement of next‐generation optoelectronic devices, particularly in applications such as high‐speed communications, precise modulation, and intelligent switching technologies.

## 5 Experimental Section

### Metamaterial Fabrication

The detailed fabrication process of the exciton‐photon coupled microcavity metamaterials can be divided into some principal steps. Primarily, a polyimide film exhibiting excellent optical transmittance was uniformly spin‐coated onto a meticulously cleaned quartz substrate, followed by baking at a carefully controlled temperature to ensure optimal adhesion and uniformity of the film. Subsequently, two layers of photoresist were sequentially applied to the polyimide film through spin‐coating, after which the substrate was precisely aligned with the mask on the photolithography machine before undergoing exposure to ultraviolet light. Following this exposure, the substrate was placed in a magnetron sputtering system, where the evaporation deposition of aluminum was carried out, resulting in aluminum structural units, thereby facilitating the patterning and preparation of the metamaterials. Finally, a monolayer graphene was transferred onto the metamaterials using a wet transfer method, completing the fabrication of the exciton‐photon coupled microcavity metamaterials.

### Bioassay Preparation

tPSA Antigen (CAS: L2C001) and Anti‐tPSA (CAS: L1C00401) were purchased from Shanghai Linc Bio‐Technology Co., Ltd. (https://www.linc‐bio.com/). In the experiment, serum purchased was used from Sigma Aldrich (CAS: F0193) as the solvent, and then continuously diluted the target antigen sample to be tested. The solutions were thoroughly mixed using a vortex mixer to prepare five groups of target antigen solutions with different concentrations (detailed information regarding the solutions is provided in Section S7, Supporting Information). In the experiment, 10 µL of each group of target antigen solution was tooked with a pipettor, and fixed the height of the pipettor to ensure the consistency of the sample area deposited on the metamaterial. To achieve greater reliability, all experimental data provided in this work are averaged over three tests.

## Conflict of Interest

The authors declare no conflict of interest.

## Supporting information



Supporting Information

## Data Availability

The data that support the findings of this study are available from the corresponding author upon reasonable request.
