# Supplementary material for: Exciton‐Photon Coupling Microcavity as a Selective Biosensing Platform for Nonlocal Terahertz Metamaterials
Source: Adv Sci (Weinh). 2025 Feb 28;12(16):2416951. doi: 10.1002/advs.202416951 (PMC12021031; doi:10.1002/advs.202416951)
Supplement: Supplementary file 1 — Supporting Information [file ADVS-12-2416951-s001.docx]

**Supporting Information**

**Exciton-Photon Coupling Microcavity as a Selective Biosensing Platform for Non-Local Terahertz Metamaterials**

G. F. Wu, F. P. Yan*, L. J. Liang*, X. M. Du, W. Wang*, T. Li, J. P. Tian, X. Yan, H. Y. Yao, Z. Q. Wang*, M. Wang

**Table of Contents**

[S1. The simulation analysis and detailed fabrication process of the hybrid metamaterial. 1](#_Toc183186914)

[S2. The experimental and simulation characterization of the hybrid metamaterials. 3](#_Toc183186915)

[S3. The detailed process of functionalized colloidal gold binding to the antibody and target tPSA 5](#_Toc183186916)

[S4. The analysis of the mechanism of the Anapole resonance generated by the metamaterial. 6](#_Toc183186917)

[S5. The strong coupling between the photonic cavity of the hybrid metamaterial and the excitons. 9](#_Toc183186918)

[S6. The optical response of the hybrid metamaterial system both before and after coupling. 11](#_Toc183186919)

[S7. Sample preparation and measurement methods 12](#_Toc183186920)

[S8. The Raman spectra of graphene in the hybrid metamaterial when detecting target tPSA at different concentrations 13](#_Toc183186921)

[S9. The phase change of monolayer graphene hybrid metamaterial biosensors 14](#_Toc183186922)

[S10. Relationship between phase and amplitude based on the KK relation 15](#_Toc183186923)

[S11. The transition from strong coupling to weak coupling. 18](#_Toc183186924)

[S12. The mechanism model of the biosensing for the hybrid metamaterial 19](#_Toc183186925)

[S13. ELISA test for benchmarking 20](#_Toc183186926)

[S14. The standardized two-dimensional wavelet coefficient intensity information corresponding to each concentration 21](#_Toc183186927)

[Reference 23](#_Toc183186928)

**Keywords:** Anapole metamaterials; exciton-photon coupling microcavity; colloidal gold; selective recognition

**S1. The simulation analysis and detailed fabrication process of the hybrid metamaterial.**

The mechanism for generating strong coupling between excitons and photons was determined by performing numerical simulations using COMSOL Multiphysics (version 6.0). In the simulation, periodic Floquet conditions were set in free space along the *x* and *y* directions, and the perfectly matched layers were used in the top and bottom domains. The proposed hybrid metamaterials were vertically illuminated by a THz wave with an electric field along the *y*-direction (*E_y_*) and a magnetic field along the *x*-direction (*H_x_*). The metamaterial microstructures were made of lossy aluminum metal, and the conductivity was set to be $\sigma=3.56\times10^{7}$S/m. Polyimide was used as the flexible substrate, and the dielectric constant and tangent loss were 3.1 and 0.05, respectively. Convergence results were obtained using a physical field grid. Additionally, graphene was modeled as a conductive surface using transition boundary conditions, with an effective thickness of 1 nm. The conductivity 𝜎(𝜔) of graphene consists of intra-band and inter-band contributions, which reflect the movement of charge carriers within the energy bands and the transitions between different energy bands, respectively. We can accurately determine the conductivity of graphene using the Kubo formula ^[1-4]^, which considers the effects of temperature, Fermi level, and various other factors.

Where $k_{B}$, , and *e* represent the Boltzmann constant, reduced Planck constant, and electronic charge, respectively. Further, *T* is the temperature (300 K), while 𝜔 and $E_{F}$ denote the angular frequency of the incident light and the Fermi energy of graphene, respectively. According to the Pauli exclusion principle, inter-band conductivity can be disregarded in the THz band ($E_{F}$≫ ℏ𝜔), and the condition $E_{F}\geq k_{B}T$is fulfilled at room temperature. Consequently, the conductivity of graphene can be expressed using the following formula ^[5, 6]^:

The effective permittivity of graphene can be calculated by $\sigma\left(\omega\right)=1+i\sigma\left(\omega\right)/\varepsilon_{0}\omega t_{g}$, where 𝜔_0_ is the vacuum dielectric permittivity.

In addition, the experimental setups mainly consist of a homogenizer (model: G3P-8), a photolithography machine (brand name: ABM), a magnetron sputter coater (brand name: Denton), and a THz time-domain spectrometer (model: TAS7500). The preparation of the metamaterial samples was carried out by a conventional photolithography and sputter coating process, and the main processing steps are shown in **Figure S1a**.

1. A polyimide film was spin-coated on a thoroughly cleaned SiO_2_ substrate;
2. Ultraviolet exposure was performed using an ABM photolithography machine, followed by development with a developer solution;
3. A Denton magnetron sputtering system was used to sputter-deposit the metal layer;
4. The sample was immersed in acetone solution for 10 minutes and then rinsed with deionized water to remove any remaining metal and photoresist;
5. Finally, a 1.0 cm × 1.0 cm monolayer of graphene was transferred to the top of the Al structural units and baked at 100°C for 30 min, completing the the fabrication of the hybrid monolayer graphene metamaterial with good uniformity.

The sample was placed on a sample stage as shown in **Figure S1b**, and the THz wave was incident vertically on the surface of the sample. The angle of incidence may be slightly off, but due to the high stability and accuracy of the system's testing, the error in angle is always kept within the normal range.


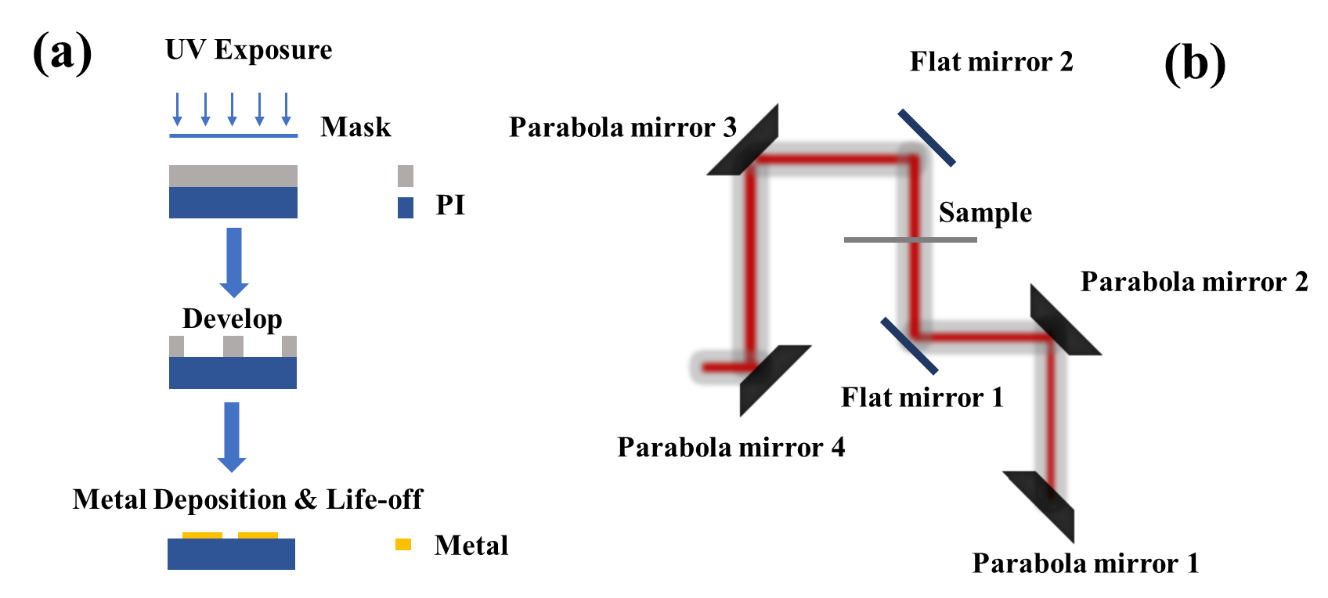


**Figure S1.** The preparation and testing of the hybrid metamaterials. (a) The patterning process of metamaterials. (b) Schematic diagram of the optical path of the test samples in the experiment.

**S2. The experimental and simulation characterization of the hybrid metamaterials.**

**Figure S2a** displays a microscope image of the fabricated structure of the metamaterials based on Anapole resonance, presenting symmetric open resonant units with well-defined shapes and clear edges. The proposed metamaterials are structured around a unit structure, which consists of two aluminum open rectangular resonant rings with a thickness of 300 nm and a periodicity of 120 µm. The geometric parameters of element structure: *a* = 100 μm, *b* =80 μm, *c* = 36 μm, *d* = 8 μm, and *e* = 12 μm. To evaluate the sensing performance of the metal-monolayer graphene hybrid metamaterial we designed, we investigated the transmission spectra of graphene under different doping levels, which correspond to varying Fermi levels, as illustrated in **Figure S2b**. It can be observed that when the Fermi level of monolayer graphene shifts from -0.1 eV to -0.3 eV, a significant redshift occurs in the transmission peak. The Rabi frequency, defined as $\Omega=\omega_{+}-\omega_{-}$, decreases from 0.276 THz to 0.263 THz. This indicates that the Rabi frequency of the monolayer graphene metamaterial diminishes as the Fermi level shifts from -0.1 eV to -0.3 eV. Due to the challenges in accurately measuring the doping condition of graphene after transfer in the experiment, we compared the simulation results with the experimental data to determine the doping status of monolayer graphene. Furthermore, there is a large radiation loss due to the high carrier concentration and conductivity of graphene, as depicted in **Figure S2c**, which attenuated the amplitude of the transmission curve with an insignificant frequency shift. The transmission curves obtained from experiments and simulations of the proposed hybrid metamaterial matched well; the slight differences can be attributed to errors in fabricating the polyimide and metal structure. Through the comparison, we can conclude that the Fermi level of graphene after transfer is approximately -0.1 eV, indicating that the monolayer graphene is in a p-type doping state at this point.

In this experiment, 20 μL of colloidal gold solution was droppd to the surface of the exciton-photon cavity hybrid sensor. After being modified with colloidal gold, the transmission curves obtained from both the experiments and the simulations of the proposed hybrid metamaterial showed good agreement, as illustrated in **Figure 2d**. In the experiment, the transmission curve of the exciton-photon hybrid cavity metamaterial is only half of that observed in the simulation. This discrepancy can primarily be attributed to the significant radiation losses caused by the high carrier concentration and conductivity of graphene. These radiation losses are more pronounced in the experiment than in the idealized model used in the simulation, resulting in further attenuation of the transmission signal. Subsequently, different concentrations of the target antigen-tPSA solution were dropped onto the surface of the colloidal gold biosensor labeled with the target antibody. Additionally, the inset in **Figure 2d** displays scanning electron microscope (SEM) images of the modified colloidal gold and the immunosensor after the binding of the target antibody to the antigen. It is important to emphasize that the total amount of tPSA antigen on the metal surface is not directly related to the presence or absence of colloidal gold, as the colloidal gold particles are retained on the monolayer graphene layer of the biosensor, where they bind with the target antibody to form complexes. This process results in a greater accumulation of tPSA antigens on the surface of the immunosensor.

**Figure S2.** The experimental and simulation characterization of the hybrid metamaterials. (a) Microscopic image of metamaterial array under an optical microscope, and the geometric parameters of element structure: *a* = 100 μm, *b* =80 μm, *c* = 36 μm, *d* = 8 μm, and *e* = 12 μm. (b) The transmission spectra of graphene under different doping levels, which correspond to varying Fermi levels. (c) The transmission spectra of the graphene metamaterial without colloidal gold. (d) After being modified with colloidal gold, the transmission curves obtained from both the experiments and the simulations of the proposed graphene metamaterial. Inset: The scanning electron microscope (SEM) images of the modified colloidal gold and the immunosensor after the binding of the target antibody to the antigen.

**S3. The detailed process of functionalized colloidal gold binding to the antibody and target tPSA**

To enable the exciton-photon hybrid metamaterial sensor to selectively recognize the target antigen-tPSA, surface functionalization of the biosensor is required. The construction steps for the hybrid sensor modified with colloidal gold-antibody probes are as follows.

(1) The hybrid metamaterial biosensor was cleaned with deionized water and alcohol, and then dried with nitrogen gas. Subsequently, a 2% solution of 3-aminopropyltriethoxysilane was added to activate the hybrid sensor, and any unbound solution was washed away using 99% anhydrous ethanol.

(2) Next, 20 μL of colloidal gold solution was added to the surface of the hybrid biosensor and incubated at 4°C for approximately 1 hour.

(3) A 10 μL solution of prostate antibody at a concentration of 20 µg/mL was uniformly applied to the sensor surface and incubated at 25°C for about 30 minutes. Subsequently, the unbound antibodies were gently rinsed off with PBS buffer 2-3 times.

(4) To block the unoccupied colloidal gold sites not bound to the prostate antibody, a 1 wt% BSA solution was added to the surface of the hybrid biosensor, followed by rinsing off the unbound BSA with buffer solution.

(5) A 10 μL solution of tPSA was then added to the surface of the hybrid biosensor and allowed to incubate at room temperature; afterward, the unbound prostate antigens were gradually washed away with buffer solution.

(6) Finally, the functionalized colloidal gold immunosensor, marked with the target antibody-antigen, was tested using a THz time-domain spectroscopy system.

**S4. The analysis of the mechanism of the Anapole resonance generated by the metamaterial.**

When the proposed metamaterial is illuminated vertically by a THz wave, with the magnetic field along the *x*-direction and the electric field along the *y*-direction at 0.98 THz, mirrored currents are established and circulate within the split resonant ring (**Figure S3a)**. These circulating currents generate magnetic dipoles (MDs) that are perpendicular to the xy-plane, extending outward in the right rectangular resonator and inward in the left rectangular resonator, thus creating oscillating loop magnetic fields. This configuration of currents is a distinctive characteristic of anapoles, resulting in reduced far-field radiation and increased near-field intensity within the split gaps. Additionally, the computed surface charge distributions and magnetic field in the z-direction at 0.98 THz are illustrated in **Figures S3b-c**. The electric dipole (ED) moment along the -y-axis is produced due to the disparity in oscillating charges accumulated in the upper and lower sections. A pair of counter-directed MDs are excited in the SRR, leading to a time-dependent (TD) moment along the +y-axis. Furthermore, the scattering powers of different multipoles are calculated by Eq. 3-7 through the spatial current density distribution based on the Cartesian coordinates to further analyze the roles of TD and ED at the observed resonance ^[7-10]^.

$$\overrightarrow{P_{x}}=\frac{1}{i\omega}\int d^{3}r\overrightarrow{j}$$

$$\overrightarrow{M}_{z}=\frac{1}{2c}\int d^{3}r(\overrightarrow{r}\times\overrightarrow{j})$$

$$Q_{m}=\frac{1}{3c}\int d^{3}r\left[\left(\overrightarrow{r}\times\overrightarrow{j}\right)_{x}r_{z}+(\overrightarrow{r}\times\overrightarrow{j})_{z}r_{x}\right]$$

where *T_y_*, *P_x_*, *M_z_*, *Q_m_*, and *Q_e_* represented the TD, ED, MD, magnetic quadrupole, and electric quadrupole, respectively. The symbols *ω* and *c* represent the angular frequency and the speed of light in a vacuum, respectively. Furthermore, the scattered power observed in the far field can be determined from the polar moments of the various multipoles, as described in Eq. 8-12.

$$I_{T}=\frac{2\omega^{6}}{3c^{5}}\left|\overrightarrow{T}_{y}\right|^{2}$$

$$I_{M}=\frac{2\omega^{4}}{3c^{3}}\left|\overrightarrow{M}_{z}\right|^{2}$$

$$I_{p}=\frac{2\omega^{4}}{3c^{3}}\left|\overrightarrow{P}_{x}\right|^{2}$$

$$I_{Qm}=\frac{\omega^{6}}{20c^{5}}\left|Q_{m}\right|^{2}$$

$$I_{Qe}=\frac{\omega^{6}}{5c^{5}}\left|Q_{e}\right|^{2}$$

where *I_T_*, *I_M_*, *I_P_*, *I_Qm_*, and *I_Qe_* are the scattering powers of the TD, ED, MD, magnetic quadrupole, and electric quadrupole, respectively. The contribution of octopoles and higher-order multipoles can be disregarded due to their negligible effect on the scattered power. Since the ED can emit the same angular momentum as the TD, the total magnetic and electric fields resulting from the combination of these two dipoles are expressed in Equations 13 and 14.

$$H_{total}=H_{p}+H_{T}=\frac{ikD(\omega,r)}{cr}\left[r\times(P-ikT)\right]\frac{e^{i(\omega t-kr)}}{r}$$

where *D*, *F*, and *G* can be consulted in ^[8, 9]^. The contributions of TD and ED predominantly govern the far-field scattered power from the metamaterials at 0.98 THz. Simultaneously, ED and TD exhibit opposite phases with a phase difference of π/2, such that destructive interference between the TD and ED leads to a nonradiating anapole resonance, as depicted in **Figures S3d-e**. This anapole principally exhibits the localization and enhancement of the electromagnetic field without the accompanying energy loss of electromagnetic radiation, and it can significantly enhance the efficiency of strong coupling with the previously mentioned excitons.

**Figure S3** The mechanism of the anapole resonance generated by the proposed hybrid metamaterials. (a) The simulated surface current distribution at 0.98 THz. (b) The proposed hybrid metamaterials, producing an electric dipole moment along the -*y*-axis. (c) The hybrid metamaterials, forming a toroidal dipole moment along the +*y*-axis. (d) The far-field scattering power of different multipoles; ED and TD have the same scattering power at 0.98 THz. (c) P and T in opposite phases with a phase difference of π/2 at 0.98 THz.

**S5. The strong coupling between the photonic cavity of the hybrid metamaterial and the excitons.**

The strong coupling between the photonic cavity of the hybrid metamaterial and the excitons enables the excitation of hybrid polariton modes, which can be modeled by diagonalizing the Hamiltonian of the coupled system ^[11-14]^.

Where $E_{B}$ and $\kappa$ represent the eigenstate energy and linewidth of the photonic modes of the hybrid metamaterial when uncoupled, while $E_{EXC}$ and γ denote the eigenstate energy and linewidth of the excitons in the uncoupled state. *g* is the coupling strength during the interaction between photons and excitons. αand β are the Hopfield coefficients used to describe the weights of the excitons and photons, satisfying α+β=1. $E_{LB}$and $E_{LB}$ represent the energy of the lower branch (LB) and upper branch (UB) of exciton-polaritons, respectively, and the expressions for these two energies can be derived from Equation 15 ^[15, 16]^.

When the detuning $\Delta=E_{B}-E_{EXC}=0$ between the photons and excitons, Equation 16 can be transformed to:

$$E_{LB,UB}=\frac{1}{2}\left[E_{EXC}+E_{B}+i\left(\gamma+\kappa\right)\right]+\sqrt{g^{2}-\frac{1}{4}\left(\gamma-\kappa\right)^{2}}$$

At this point, the energy difference corresponds to the value of Rabi splitting:

Rabi splitting is an inevitable result of the strong coupling between excitons and photons; therefore, the conditions for achieving strong coupling can be derived from Equation 18 as follows:

$$g>\frac{\gamma-\kappa}{2}$$

To achieve separation of the two modes in the transmission spectrum, the minimum splitting value must be greater than the sum of the linewidths of the two resonances. Therefore, the following conditions must also be satisfied for strong coupling ^[17, 18]^:

or $\text{g}>\sqrt{\left(\gamma^{2}+\kappa^{2}\right)/2}$

To ascertain whether a system has entered the strong coupling regime, it is crucial not only to analyze the anti-crossing behavior in its absorption spectrum but also to evaluate the relationship between the coupling strength of the excitons and photons and the dissipation modes. This assessment is necessary to determine if the conditions for strong coupling are satisfied. According to the definition in Equation 18, the value of the Rabi splitting can be directly obtained from **Figure 2b** in the manuscript (red solid line) as = 1.323 meV. From the Fano fitting of the blue and green solid lines in **Figure 2b**, the linewidths of the excitons and photons are calculated to be *γ* = 0.39 meV and *κ* = 0.875 meV, respectively. The coupling strength ggg can be calculated from Equation 18 as *g* = 0.705 meV. These results are in substantial agreement with those derived from direct fitting of the simulated spectra, indicating that the exciton-photon coupling falls within the strong coupling regime.

**S6. The influence of structural parameters on the Rabi splitting phenomenon in the hybrid metamaterial.**

The phenomenon of anti-crossing and Rabi splitting observed in both simulations and experiments arises from the strong coupling effects between the exciton and anapole resonance modes in metamaterials. Specifically, Rabi splitting, a hallmark of strongly coupled systems, describes the energy difference generated between two distinct modes due to their coupling, with this energy difference corresponding to the separation between two transmission peaks. Theoretically, this phenomenon can be accurately described using a coupled Lorentz oscillator model. This model is based on the interaction between two oscillatory modes: the plasmonic resonance mode supported by graphene (oscillator 1) and the non-radiative anapole resonance (oscillator 2), with the latter being driven by the incident electric field of electromagnetic waves. Consequently, the resonant amplitudes of the two oscillators (*x₁*(*t*) and *x₂*(*t*)) can be described by the coupled Equations 21 and 22 ^[19, 20]^.

(21)

(22)

where *ω₀* represents the resonant frequency of oscillator 1, while *δ* and *κ* correspond to the detuning of the resonant frequency and the coupling coefficient between the two oscillators, respectively. *γ₁* and *γ₂* are the damping rates of the two oscillators, and a denotes the coupling coefficient between the broad oscillator 1 and electric field. By applying the approximation$\omega-\omega_{0}\ll\omega_{0}$, and solving Equations 21 and 22, the susceptibility *χ* can be derived as follows ^[19]^:

$\chi=\chi_{r}+i\chi_{i}\propto\frac{(\omega-\omega_{0}-\delta)+i\frac{\gamma_{2}}{2}}{(\omega-\omega_{0}+i\frac{\gamma_{1}}{2})(\omega-\omega_{0}-\delta+i\frac{\gamma_{2}}{2})-\frac{\kappa^{2}}{4}}$ (23)

where *χᵣ* and *χᵢ* represent the real and imaginary components of *χ*, respectively. As the energy dissipation is in proportion to *χ_i_*, the transmittance *T* can therefore be expressed as:

$T=1-\alpha\chi_{i}$ (24)

Using this formula, we can predict the transmission characteristics of the hybrid metamaterial, as shown in **Figure S4a**. The fitted curve aligns remarkably well with the simulation results, with only a slight deviation attributed to the periodic effects of the hybrid metamaterial ^[20]^. Moreover, the energy states of excitons and photons transition from being simple independent states to forming hybrid states, resulting in energy-level interleaving rather than mere crossing. The new energy states behave as two separated energy branches — specifically, two distinct transmission peaks (upper polariton branch (UPB) and lower polariton branch (LPB)), which are “anti-crossing” in energy–momentum space. The geometric parameters, such as the thickness of the aluminum metal and the period of the resonant units, exert different influences on the non-radiative anapole resonance and the exciton. In this context, by employing the coupled model, we describe the impact of the metal thickness on the interaction characteristics between the proposed graphene plasmonic mode and the anapole resonance, as expressed by the following equation ^[19, 20]^:

$E_{\pm}=(E_{OD}+E_{MP})/2\pm\sqrt{g^{2}+(E_{OD}-E_{MP})^{2}/4}$ (25)

where *E_OD_* and *E_OP_* represent the resonant energies of the non-radiative anapole mode and the fundamental plasmonic mode, respectively, while the coupling strength is indicated by the Rabi splitting energy (*g*). Thus, we performed numerical simulations of the transmission spectra for the exciton, photon, and their coupled system within a thickness range of 0.1 μm to 0.35 μm, as shown in **Figure S4b**. The results show that, as the system thickness increases, the position of the exciton (red solid line) remains unchanged. This suggests that the optical properties of the exciton material itself are not significantly affected by the thickness variation, and it maintains its original energy level. By fitting the Equation 23, the resonant frequencies of the two peaks, *ω-* and *ω_+_*​, at different metal thicknesses can be accurately predicted, as illustrated by the two blue branches in **Figure S4b**. Although the phenomenon of Rabi splitting in the hybrid system remains pronounced with increasing thickness, the dispersion curve of the anapole resonance exhibits a linear relationship with thickness, showing a clear redshift. This is because the increase in thickness alters the effective refractive index of the metamaterial, causing the resonance frequency of the anapole mode to shift to lower energies. Additionally, to further explore this dispersion behavior, we plotted a 2D image of the transmission spectra as a function of thickness, clearly observing the appearance of anti-crossing behavior (**Figure S4c**). The results reveal that at the point where the plasmonic resonance intersects with the non-radiative anapole resonance, the two transmission peaks (*ω-* and *ω_+_​*) exhibit a distinct anti-crossing phenomenon, unveiling the intriguing Rabi splitting effect, which provides crucial evidence for the strong coupling between the two resonant modes.

**Figure S4.** The influence of structural parameters on the Rabi splitting phenomenon in the hybrid metamaterial. (a) The transmission spectrum of the hybrid metamaterial from simulation and theoretical predictions. (b) Optical response before and after system coupling: The black dots and blue solid line represent the two energy levels of the sub-polariton modes, obtained from numerical simulations and theoretical fitting, respectively. The red line corresponds to the exciton resonance mode in the uncoupled state, while the green line represents the anapole resonance mode in the uncoupled state. (c) The dispersion behavior of the exciton polariton in the transmission spectrum.

**S7.** **Sample preparation and measurement methods**

To verify the sensing performance of the designed hybrid metamaterial and its selective detection capability for target antigens, we prepared different concentrations of α-Lactalbumin, D-Threonine, L-Arginine, target antigen-tPSA, and target antibody solutions as the detection objects. α-Lactalbumin (≥ 90%, CAS: 9051-29-0), D-Threonine (≥ 99%, CAS: 632-20-2), and L-Arginine (≥ 99%, CAS: 74-79-3) were purchased from MACKLIN (Shanghai, China) (<https://www.macklin.cn/>); Colloidal gold was purchased from Nanjing XFNANO Materials Tech Co.,Ltd (0.05mg mL^-1^, 15 nm in diameter); tPSA Antigen (1mg mL^-1^, CAS: L2C001) and Anti-tPSA (1mg mL^-1^, CAS: L1C00401) were purchased from Shanghai Linc Bio-Technology Co., Ltd. (<https://www.linc-bio.com/>). In the experiment, we used serum purchased from Sigma Aldrich (CAS: F0193) as the solvent, and then continuously diluted the target antigen sample to be tested. The solutions were thoroughly mixed using a vortex mixer to prepare five groups of target antigen solutions with different concentrations. In the experiment, we we took 10 μL of each group of target antigen solution with a pipettor, and fixed the height of the pipettor to ensure the consistency of the sample area deposited on the metamaterial. To achieve greater reliability, all experimental data provided in this work are averaged over three tests. Moreover, after completing all the experiments for the first biosensor, we repeated them on another biosensor prepared at the same time, using the same conditions, to prove that the results were reliable and not accidental.

**S8. The Raman spectra of graphene in the hybrid metamaterial when detecting target tPSA at different concentrations**

To verify the accuracy of our explanation regarding the interaction process between different concentrations of tPSA solution and monolayer graphene, we employed Raman spectroscopy to measure monolayer graphene on the surface of the hybrid metamaterial in various states, as shown in **Figure S5**. The G peak in the Raman spectrum of monolayer graphene is highly sensitive to doping levels and, therefore, serves as an experimental parameter for quantitatively assessing the doping level of the graphene. In the absence of the target antigen, the G peak of monolayer graphene is located at 1579.1 cm⁻¹, indicating p-type doping with the Fermi level significantly distanced from the Dirac point ^[18]^. As the tPSA protein concentration increases to C_5_, a pronounced blue shift in the G peak is observed, signaling that the Fermi level of monolayer graphene moves further from the Dirac point, thereby enhancing the p-type doping effect. The underlying cause of this blue shift lies in the unique electronic structure of monolayer graphene, which features a Dirac cone ^[5, 6]^. P-type doping lowers the Fermi level, causing electrons to fill lower energy states. As the tPSA concentration increases, tPSA molecules interact with the graphene surface, further elevating the carrier concentration in the graphene. This change leads to the generation or rearrangement of electron-hole pairs (e-h pairs), altering the concentration and distribution of carriers, which subsequently impacts the interaction between phonons and electrons. Moreover, the optical properties of monolayer graphene are intricately linked to the surface carrier concentration. As the carrier concentration increases, the scattering rate of carriers in the graphene may also rise, resulting in enhanced absorption of THz waves, which, in turn, reduces the transmittance.

**Figure S5.** The Raman spectra of graphene in the hybrid metamaterial when detecting target tPSA at different concentrations.

**S9. The phase change of monolayer graphene hybrid metamaterial biosensors**

As the concentration increases from C_0_ (bare) to C_5_, the coupling mechanism between photons and excitons in the immunosensor undergoes a noticeable change. The enhancement of the local electric field on the sensor surface directly affects the propagation characteristics of light, ultimately leading to a significant increase in the phase variation of the THz waves. Specifically, as the concentration rises, the hybrid metamaterial immunosensor achieves a phase difference of 210.62° at the coupling frequency point (*ω_+_*) relative to the bare curve. Moreover, we found that the phase change in the THz metamaterial biosensor labeled with antibodies and modified with colloidal gold was significantly larger than in sensors that were either unlabeled with antibodies or unmodified with colloidal gold, as shown in **Figure S6**. This is primarily due to the significant enhancement of the coupling between photons and excitons by antibodies and colloidal gold, which generates a stronger local electric field amplification, directly improving the optical response on the sensor surface. Additionally, antibody labeling allows the sensor surface to specifically recognize target molecules, inducing changes in both electrical and optical properties, thereby further amplifying the phase shift. At the same time, the plasmonic resonance properties of colloidal gold increase the optical active region on the sensor surface, making the phase response more sensitive and pronounced, ultimately resulting in a larger phase shift.

**Figure S6.** The phase change of monolayer graphene hybrid metamaterial biosensors in detecting different concentrations of tPSA proteins is presented, including sensors that are unlabeled with anti-tPSA, unmodified with colloidal gold, and simultaneously labeled with anti-tPSA and modified with colloidal gold. (a) The biosensor is unlabeled with anti-tPSA. (b) The biosensor is unmodified with colloidal gold. (c) The immunosensor.

**S10. Relationship between phase and amplitude based on the KK relation**

The Kramers-Kronig (KK) relation establishes a connection between the real and imaginary components of the causal response function $G\left(t\right)$ that describes the electromagnetic wave function. The electromagnetic wave function is essentially a frequency-domain response function, represented by a complex relationship: $G\left(\omega\right)\text{ }=\text{ }G'\left(\omega\right)\text{ }+\text{ }iG''\left(\omega\right)$, where $G'\left(\omega\right)$ and $G''\left(\omega\right)$ are the real and imaginary components of the function, respectively, as shown in Equation 26 ^[21-23]^.

$\begin{matrix} G'(\omega)=\frac{2}{\pi}P\int_{0}^{\infty} \frac{\omega'G"(\omega')}{\omega'^{2}-\omega^{2}}d\omega',G"(\omega)=\frac{2\omega}{\pi}P\int_{0}^{\infty} \frac{G'(\omega')}{\omega'^{2}-\omega^{2}}d\omega' \\ \end{matrix}$ (26)

Where the *P* represents the Cauchy principal value. It is known that the amplitude and phase components of the frequency-domain response function of electromagnetic wave can be separated by Napierian loga-rithm as:

$\ln G(\omega)=\ln\left|G(\omega)\right|+i\angle G(\omega)$ (27)

where the phase part and amplitude part of the complex electromagnetic wave function are represented as $\angle G(\omega)$ and $\ln\left|G(\omega)\right|$, respectively. Through Equation 27, the expression of the relationship between phase and amplitude can be obtained:

$\angle G(\omega)=-\frac{2\omega}{\pi}P\int_{0}^{\infty} \frac{\ln\left|G(\omega')\right|}{\omega'^{2}-\omega^{2}}d\omega'$ (28)

Then, the change variables have been applied:$v=\ln(\frac{\omega'}{\omega})$, or $\omega'=\omega e^{v}$and $M(v)=\ln\left|G(\omega')\right|$, where the range of the integration $v\to(-\infty,\infty)$, $d\omega'=\omega e^{v}dv$, and the Equation 28 can then be derived as:

$\begin{matrix} \angle G(\omega)=-\frac{2\omega}{\pi}P\int_{-\infty}^{\infty} \frac{M(v)}{\omega^{2}e^{2v}-\omega^{2}}\omega e^{v}dv \\ n-\frac{2}{\pi}P\int_{-\infty}^{\infty} \frac{M(v)}{e^{2v}-1}e^{v}dv,n-\frac{1}{\pi}P\int_{-\infty}^{\infty} \frac{M(v)}{\sinh v}dv \end{matrix}$ (29)

As the sinh is an odd function, only the odd part of $M\left(v\right)$ contributes in Equation 24. $M\left(v\right)$ is the sum of odd and even function, $M\left(v\right)\text{ }=\text{ }Mo\text{ }+\text{ }Me$.

$P\int_{-\infty}^{\infty} \frac{M(v)}{\sinh v}dv=\lim_{\varepsilon\to0+} \left\{\int_{-\infty}^{-\varepsilon}+\int_{\varepsilon}^{\infty}\right\}\frac{M_{o}(v)+M_{e}(v)}{\sinh v}dv=2\lim_{\varepsilon\to0+} \int_{\varepsilon}^{\infty} \frac{M_{o}(v)}{\sinh v}dv$ (30)

Considering the relation of $\int\frac{1}{\sinh v}dv=-\mathrm{lncoth}\frac{v}{2}$ with integrating process, therefore, Equation 30 can be rewritten as:

$P\int_{-\infty}^{\infty} \frac{M(v)}{\sinh v}dv=2\lim_{\varepsilon\to0+} \left\{\int_{\varepsilon}^{\infty} \frac{dM_{o}}{dv}\mathrm{lncoth}\frac{v}{2}dv-M_{o}\mathrm{lncoth}\left.\frac{v}{2}\right|_{\varepsilon}^{\infty}\right\}$ (31)

Furthermore, assuming the boundary terms vanishing, as $v\to\infty$,$\mathrm{lncoth}\frac{v}{2}\sim2e^{-v}=2\frac{\omega}{\omega'}$. As $\omega'\to\infty,\left|G(\omega')\right|:\omega'^{-n}$, as physical response functions vanish at infinite frequencies. Then, $\ln\left|G(\omega')\right|:-n\ln\omega'$. Thus, $M_{O}\mathrm{lncoth}\frac{v}{2}\sim\frac{\ln\omega'}{\omega'}\to0$. As $v\to\infty$,$\mathrm{lncoth}\frac{v}{2}\approx-\ln\frac{v}{2}$. As $M_{o}$ is odd, $M_{o}\sim v+O(V^{3})$. Thus, $v\ln v\to0$, and Equation 29 can be rewritten as:

$\angle G(\omega)=-\frac{2}{\pi}\int_{0}^{\infty} \frac{dM_{o}}{dv}\mathrm{lncoth}\frac{v}{2}dv=-\frac{1}{\pi}\int_{-\infty}^{\infty} \frac{dM}{dv}\mathrm{lncoth}\frac{\left|v\right|}{2}dv$ (32)

Finally, the phase function can be expressed as a relation of the derivative of the amplitude function, as shown in Equation 33. This relationship indicates that the change in phase is closely related to the variation in the amplitude function, and the phase change trend can be precisely described through the derivative of the amplitude function.

$\begin{matrix} \angle G(\omega)=\frac{\pi}{2}\int_{-\infty}^{\infty} \frac{dM_{O}}{dv}f(v)dv,f(v)=-\frac{2}{\pi^{2}}\mathrm{lncoth}\frac{\left|v\right|}{2},M(v)=\ln\left|G(\omega')\right| \\ \end{matrix}$ (33)

Around the resonant frequency peak, $v\to0,\omega\to\omega',dM_{o}/dv=d\ln\left|G\right|/d\ln\left|\omega\right|$, so that the phase is positively correlated with the derivative of the amplitude function. According to the KK relation, a phase jump inevitably occurs near the transmission resonance peak, with stronger resonance intensity leading to a more pronounced phase change. For instance, in the hybrid metamaterial immunosensor at a concentration of C_5_ = 1 μg mL^-1^, the maximum phase changes (B′, B″, and B‴) correspond to the largest $d\ln\left|G\right|/d\ln\left|\omega\right|$ (A′, A″, and A‴), and the $\ln\left|G(\omega)\right|$ is just the amplitude function (the transmission function). Variations in the amplitude function not only determine the strength of the optical response but also influence the magnitude and sensitivity of the phase jump. Consequently, theoretical analyses and experimental results consistently affirm that phase shifts are directly linked to the amplitude function. This further underscores the significance of transmission resonance characteristics in designing high-performance sensors, as illustrated in **Figure S7**.

**Figure S7.** A phase jump inevitably occurs near the transmission resonance peak, with stronger resonance intensity leading to a more pronounced phase change.

**S11. The transition from strong coupling to weak coupling.**

The variation in the Rabi splitting value is also affected by the energy difference between the excited and ground states of the excitons. As the concentration of target tPSA protein increases, the molecular adsorption effect becomes more pronounced, leading to an enhanced electron shielding effect, which further reduces the density of states of excitons and decreases the coupling efficiency between photons and excitons. Additionally, the increased molecular scattering and absorption disrupt the energy exchange between excitons and photons, resulting in a reduction in the coupling strength *g*. Most critically, the energy difference between the exciton’s excited state and ground state may shift due to changes in the environment, affecting the Rabi splitting value. With the continuous increase in the concentration of the analyte, these factors collectively lead to a transition of the coupling system from strong coupling to weak coupling, manifested by the gradual decline in coupling strength and Rabi splitting value until $\text{g < }\left[\left(\gamma^{2}+\kappa^{2}\right)/2\right]^{0.5}$, as depicted in **Figure S8**. This process vividly illustrates the highly sensitive response mechanism of the immunosensor to variations in the target molecular concentration, providing a theoretical foundation for high-precision biological and chemical sensing.

**Figure S8.** The transition from strong coupling to weak coupling.

**S12. The mechanism model of the biosensing for the hybrid metamaterial**

The physical mechanism of frequency shifts in the hybrid metamaterial biosensing platform can be qualitatively explained using perturbation theory. In electromagnetics, the two curl equations related to the electric and magnetic fields (*E*_AM_(*r*) and *H*_AM_(*r*)) of the original metamaterial can be reformulated, such that under the conditions of the electric field, they are expressed as ^[5, 6]^:

$\nabla\times\nabla\times E_{\text{AM}}\left(r\right)=\left(\frac{\omega_{\text{AM}}}{c}\right)^{2}\varepsilon\left(r\right)E_{\text{AM}}\left(r\right)$ (34)

We analyzed the relationship between the Δƒ value and changes in the dielectric environment using a modified perturbation theory. The relative change in angular frequency, Δω_AM_, can be calculated using the following formula:

$\frac{\Delta\omega_{\text{AM}}}{\omega_{\text{AM}}}=-\frac{1}{2}\frac{\int_{0}^{h} E_{\text{AM}}\left(r\right)\cdot\left(\varepsilon-1\right)\cdot E_{\text{AM}}\left(r\right)dr}{\int_{0}^{\infty} \left(\left|E_{\text{AM}}\left(r\right)\right|^{2}\right)dr}$ (35)

where *ε* represents the equivalent dielectric constant of the hybrid metamaterial. Since the electric field decays exponentially along the direction perpendicular to the metamaterial, the frequency shift calculated using Equation 35 has the following relationship with the amount of analyte:

$\frac{\Delta\omega_{\text{AM}}}{\omega_{\text{AM}}}\propto\frac{\left(\varepsilon-1\right)\cdot h}{l}=\Delta\varepsilon\cdot\frac{h}{l}\propto\Delta\varepsilon$ (36)

where Δ*ε* is the difference in the equivalent dielectric constant with and without the target antigen, *h* is the thickness of the monolayer graphene (approximately 1 nm), and *l* is the penetration depth of the THz field. It can be concluded that the introduction of the target antigen causes a change in the conductivity of the monolayer graphene which alters the dielectric environment of the hybrid metamaterial, leading to an increase in Δ*f* as the concentration increases.

**S13.** **ELISA test for benchmarking**

We have conducted an ELISA test for benchmarking the performance of the proposed exciton-photon coupled cavity-based hybrid metamaterial for detecting target antigens. The principle of the ELISA test is as follows: tPSA antibodies are immobilized onto the wells of an enzyme-linked immunosorbent assay (ELISA) plate to capture tPSA antigens present in the sample. Once the antigen binds to the antibody, biotinylated tPSA antibodies and horseradish peroxidase (HRP)-labeled streptavidin is sequentially introduced to form an immune complex. Unbound components are then removed through a washing step. Following this, a colorimetric substrate (TMB) is added, which undergoes oxidation and turns blue under HRP catalysis. The reaction is subsequently stopped, causing the color to shift to yellow. The optical density (OD) is measured at a wavelength of 450 nm. The OD_450_ value is directly proportional to the tPSA concentration in the sample, and the tPSA concentration is determined by referencing the standard calibration curve. However, due to the limitations of the ELISA kit, we could only detect concentrations in the range of 0.313 ng/mL to 20 ng/mL, as depicted in **Figure S9**. In this experiment, we observed that the ELISA kit was unable to detect lower concentrations, particularly in the femtomolar and picomolar ranges. Furthermore, ELISA measurements require a longer time and involve multiple washing steps. In contrast, the hybrid metamaterial integrated with gold colloids provides a faster and more sensitive detection method.

**Figure S9.** ELISA test for benchmarking.

**S14. The standardized two-dimensional wavelet coefficient intensity information corresponding to each concentration**

To elucidate the interaction between THz waves and the immunosensors at varying concentrations of target tPSA protein with clarity and comprehensiveness, we proposed a novel methodology that employs continuous wavelet transform (CWT) in conjunction with the convolution of Morlet wavelets applied in THz time-domain spectroscopy, and this approach enables the rapid identification of trends in concentration variations. For measured signals in the time domain, the fundamental definition of CWT can be expressed as follows ^[24-26]^:

$W_{\psi}f(a,b)=\int_{-\infty}^{\infty} f(t)\frac{1}{\sqrt{a}}\psi^{*}(\frac{t-b}{a})dt$ (37)

where *a* is a scale parameter analogous to frequency, *b* is a position parameter analogous to the positional parameter of time delay, and *Ψ*^∗^ is the complex conjugate of *Ψ*, whose equation can be expressed as:

$\psi_{a,b}(t)=\psi_{a}(t-b)=\frac{1}{\sqrt{a}}\psi(\frac{t-b}{a})$ (38)

$W_{\psi}f(a,b)=\langle f(t),\psi_{a,b}(t)\rangle$ (39)

The inner product between the wavelet function and the time-domain signal is calculated using Equation 39. This approach allows one-dimensional data to be transformed into a two-dimensional function defined by two adjustable parameters: the scale parameter *a* and the position parameter *b*. By varying *a* and *b*, wavelet coefficients corresponding to different concentrations can be derived. The complex Morlet wavelet is chosen as the primary wavelet:

$\psi(t)=\frac{1}{\sqrt{\pi f_{b}}}\cdot e^{j2\pi f_{c}t}-(t^{2}/f_{b})$ (40)

where *f_c_* and *f_b_* are, respectively, the central frequency and bandwidth of the Morlet wavelet, both selected as 3 THz. We acquire the time signals by THz time-domain spectroscopy and use Equations 39 and 40 to obtain the joint time-frequency two-dimensional signals. By processing time signals at different concentrations and analyzing the detailed interaction between THz waves and the hybrid metamaterial immunosensor under varying concentrations, standardized two-dimensional wavelet coefficient intensity maps corresponding to each concentration were obtained, as shown in **Figure 4a** (manuscript). As can be seen in the figure, the transmission amplitude of THz waves gradually diminishes as the concentration increases, and this change is not only evident in the frequency domain but also highlights the propagation characteristics of THz waves in the time domain, along with the significant impact of the coupling effects with the immunosensor within a specific time window (0 to 60 ps). This dynamic process is clearly represented in the time-frequency domain, revealing the profound influence of concentration changes on the characteristics of THz waves.

**S15. The sensing impact of monolayer graphene on hybrid metamaterials**

To gain a deeper understanding of the specific contribution of graphene to the performance enhancement, we have conducted a detailed analysis based on the experimental data presented in the study. As illustrated in **Figure S10a,** the biosensor without monolayer graphene, consisting of a surface-modified anti-tPSA colloidal gold hybrid metamaterial, is still able to detect varying concentrations of the target tPSA proteins. Notably, the maximum modulation depth (∆*T*) achieved by this system at a concentration of 1 µg/mL (C_5_) is 32.6%. This demonstrates the immunosensor's ability to detect specific biomarkers effectively, even in the absence of graphene. However, the introduction of monolayer graphene into the hybrid metamaterial significantly improves the sensor’s performance. The coupling between photons and excitons is notably enhanced when graphene is incorporated into the system. This enhancement is facilitated through resonant electromagnetic interactions that promote strong coupling between the photon and exciton modes, giving rise to the formation of polariton states. The unique properties of graphene, particularly its low-dimensional structure and high electron mobility, enable efficient coupling with the electromagnetic field, which leads to a significant increase in the sensor's sensitivity. As observed in the experimental data, the maximum modulation depths at the coupling frequency points (ω- and ω_+_) with monolayer graphene reach 81.5% and 85.4% (**Figure 3, Manuscript**), respectively, which represents a substantial improvement compared to the sensor without graphene. In the presence of graphene, the system exhibits a pronounced Rabi splitting, and the coupling strength is significantly increased, indicating that the system remains in the strong coupling regime. Furthermore, the introduction of graphene results in a substantial redshift of the coupling frequencies, with the maximum shifts of ω- and ω+ reaching 79 GHz and 99.6 GHz (**Figure 3, Manuscript**), respectively, at a concentration of C_5_. This shift is significantly larger than that observed for the hybrid metamaterial without graphene, highlighting the enhanced coupling effect enabled by the monolayer graphene. Therefore, the system with graphene maintains superior performance in terms of modulation depth, Rabi splitting, and frequency shifts, confirming the substantial improvement brought about by the incorporation of graphene into the hybrid metamaterial. The enhanced sensitivity and performance of the sensor with graphene make it a promising candidate for ultra-sensitive biosensing applications, particularly in detecting trace amounts of biomolecules, which is a critical requirement in many biomedical and diagnostic settings.

**Figure S10.** The transmission spectra of the metamaterial immunosensor for detecting different concentrations of tPSA proteins with and without monolayer graphene. (a) Without monolayer graphene. (b) With monolayer graphene.

**Reference**

[1] F. Xu, S. Das, Y. Gong, Q. Liu, H.C. Chien, H.Y. Chiu, J. Wu, R. Hui, Complex refractive index tunability of graphene at 1550 nm wavelength, Applied Physics Letters 106(3) (2015).

[2] Z. Fan, A. Uppstu, T. Siro, A. Harju, Efficient linear-scaling quantum transport calculations on graphics processing units and applications on electron transport in graphene, Computer Physics Communications 185(1) (2014) 28-39.

[3] A. Gallerati, Graphene properties from curved space Dirac equation, The European Physical Journal Plus 134(5) (2019).

[4] T.O. Wehling, S. Yuan, A.I. Lichtenstein, A.K. Geim, M.I. Katsnelson, Resonant scattering by realistic impurities in graphene, Phys Rev Lett 105(5) (2010) 056802.

[5] H. Yao, Z. Sun, X. Yan, M. Yang, L. Liang, G. Ma, J. Gao, T. Li, X. Song, H. Zhang, Q. Yang, X. Hu, Z. Wang, Z. Li, J. Yao, Ultrasensitive, light-induced reversible multidimensional biosensing using THz metasurfaces hybridized with patterned graphene and perovskite, Nanophotonics 11(6) (2022) 1219-1230.

[6] X. Yan, T. Li, G. Ma, J. Gao, T. Wang, H. Yao, M. Yang, L. Liang, J. Li, J. Li, D. Wei, M. Wang, Y. Ye, X. Song, H. Zhang, C. Ma, Y. Ren, X. Ren, J. Yao, Ultra-sensitive Dirac-point-based biosensing on terahertz metasurfaces comprising patterned graphene and perovskites, Photonics Research 10(2) (2022).

[7] W. Wang, Y.K. Srivastava, M. Gupta, Z. Wang, R. Singh, Photoswitchable Anapole Metasurfaces, Advanced Optical Materials 10(4) (2021).

[8] M. Gupta, Y.K. Srivastava, R. Singh, A Toroidal Metamaterial Switch, Adv Mater 30(4) (2018).

[9] H. Pan, H.f. Zhang, Broadband Polarization‐Insensitive Coherent Rasorber in Terahertz Metamaterial with Enhanced Anapole Response and Coupled Toroidal Dipole Modes, Advanced Optical Materials 10(2) (2021).

[10] L. Zeng, H.-F. Zhang, Absorption Improvement of the Anapole Metastructure for Sensing Applications, IEEE Sensors Journal 22(12) (2022) 11644-11652.

[11] S. Zhang, Q. Shang, W. Du, J. Shi, Z. Wu, Y. Mi, J. Chen, F. Liu, Y. Li, M. Liu, Q. Zhang, X. Liu, Strong Exciton–Photon Coupling in Hybrid Inorganic–Organic Perovskite Micro/Nanowires, Advanced Optical Materials 6(2) (2017).

[12] Z. Tahir, J.-W. Jung, M.U. Rashid, S. Kim, D.K. Dang, J.-W. Kang, C.-H. Cho, J.I. Jang, Y.S. Kim, Strong exciton-photon coupling in self-hybridized organic–inorganic lead halide perovskite microcavities, Nanophotonics 12(23) (2023) 4297-4306.

[13] Y. Zhang, E. Fan, Hamiltonian structure of the integrable coupling of the Jaulent–Miodek hierarchy, Physics Letters A 348(3-6) (2006) 180-186.

[14] W. Du, S. Zhang, Q. Zhang, X. Liu, Recent Progress of Strong Exciton-Photon Coupling in Lead Halide Perovskites, Adv Mater 31(45) (2019) e1804894.

[15] L. Zhao, Q. Shang, M. Li, Y. Liang, C. Li, Q. Zhang, Strong exciton-photon interaction and lasing of two-dimensional transition metal dichalcogenide semiconductors, Nano Research 14(6) (2020) 1937-1954.

[16] F. Muckel, K.N. Guye, S.M. Gallagher, Y. Liu, D.S. Ginger, Tuning Hybrid Exciton-Photon Fano Resonances in Two-Dimensional Organic-Inorganic Perovskite Thin Films, Nano Lett 21(14) (2021) 6124-6131.

[17] L. Yang, Excitons in intrinsic and bilayer graphene, Physical Review B 83(8) (2011).

[18] L. Xu, J. Xu, W. Liu, D. Lin, J. Lei, B. Zhou, Y. Shen, X. Deng, Terahertz metal-graphene hybrid metamaterial for monitoring aggregation of Aβ16–22 peptides, Sensors and Actuators B: Chemical 367 (2022).

[19] L. Yu, F. Ji, T. Guo *et al.*, “Ultraviolet thermally tunable silicon magnetic plasmon induced transparency,” [*Optics Communications*](https://www.sciencedirect.com/journal/optics-communications) **2025**, 575, 131312.

[20] C. Zhang, Y. Wang, Y. Yao *et al.*, “Active control of electromagnetically induced transparency based on terahertz hybrid metal-graphene metamaterials for slow light applications,” [*Optik*](https://pubs.acs.org/journal/aanmf6?ref=breadcrumb) **2020**, 200, 163398.

[21] Y. Zhang, Y. Zhao, S. Liang, B. Zhang, L. Wang, T. Zhou, W. Kou, F. Lan, H. Zeng, J. Han, Z. Feng, Q. Chen, P. Mazumder, Z. Yang, Large phase modulation of THz wave via an enhanced resonant active HEMT metasurface, Nanophotonics 8(1) (2018) 153-170.

[22] H.-T. Chen, W.J. Padilla, M.J. Cich, A.K. Azad, R.D. Averitt, A.J. Taylor, A metamaterial solid-state terahertz phase modulator, Nature Photonics 3(3) (2009) 148-151.

[23] J. Bechhoefer, Kramers–Kronig, Bode, and the meaning of zero, American Journal of Physics 79(10) (2011) 1053-1059.

[24] X. Guo, Z. Zhang, M. Yang, P. Bing, X. Yan, Q. Yang, D. Wei, L. Liu, L. Liang, J. Yao, Time-Frequency Double Domain Resolving by Electromagnetically Induced Transparency Metasensors for Rapid and Label-Free Detection of Cancer Biomarker Midkine, Optics and Lasers in Engineering 142 (2021).

[25] Z. Zhang, J. Gao, M. Yang, X. Yan, Y. Lu, L. Wu, J. Li, D. Wei, L. Liu, J. Xie, L. Liang, J. Yao, Microfluidic integrated metamaterials for active terahertz photonics, Photonics Research 7(12) (2019).

[26] C. Huang, L. Liang, P. Chang, H. Yao, X. Yan, Y. Zhang, Y. Xie, Terahertz Liquid Biosensor Based on A Graphene Metasurface for Ultrasensitive Detection with A Quasi-Bound State in the Continuum, Adv Mater 36(11) (2024) e2310493.
